# Spin-orbital effects in metal-dichalcogenide semiconducting monolayers

**DOI:** 10.1038/srep24093

**Published:** 2016-04-20

**Authors:** J. A. Reyes-Retana, F. Cervantes-Sodi

**Affiliations:** 1 Universidad Iberoamericana, Departamento de Física y Matemáticas, Prolongación Paseo de la Reforma 880, Lomas de Santa Fe, Mexico City, 01219, México

## Abstract

Metal-dioxide & metal-dichalcogenide monolayers are studied by means of Density Functional Theory. For an accurate reproduction of the electronic structure of transition metal systems, the spin orbit interaction is considered by using fully relativistic pseudopotentials (FRUP). The electronic and spin properties of MX_2_ (M = Sc, Cr, Mn, Ni, Mo & W and X = O, S, Se & Te) were obtained with FRUP, compared with the scalar relativistic pseudopotentials (SRUP) and with the available experimental results. Among the differences between FRUP and SRUP calculations are giant splittings of the valence band, substantial band gap reductions and semiconductor to metal or non-magnetic to magnetic “transitions”. MoO_2_, MoS_2_, MoSe_2_, MoTe_2_, WO_2_, WS_2_ and WSe_2_ are proposed as candidates for spintronics, while CrTe_2_, with *μ* ~ 1.59 *μ*_*B*_, is a magnetic metal to be experimentally explored.

The synthesis of single layer graphene in 2004^1^ has been the trigger for a colossal amount of studies that uncovered the novel physical properties present in two dimensional (2D) materials^2^,^3^,^4^,^5^,^6^,^7^, which in turn evolved in a complete new branch of theoretical and experimental research within condensed matter physics^8^,^9^,^10^,^11^,^12^,^13^,^14^.

These works have led to significant advancements of emerging technologies with 2D materials^6^,^8^ such as: micro and nanoelectronics^1^,^15^,^16^,^17^, sensing^18^,^19^, energy storage^20^,^21^, energy conversion^22^,^23^,^24^, photonics^25^,^26^, optoelectronics^7^, magnetoresistance^27^ and spintronics/valleytronics^28^,^29^,^30^,^31^; motivating the search for new 2D semiconducting materials.

The effort to isolate different layered materials started almost simultaneous to the first isolation of single layer graphene^3^. The mechanical cleavage of MoS_2_ and NbSe_2_ 2D crystals opened the research towards quasi-two dimensional transition-metal dichalcogenides (2D-MX_2_)^32^, materials with a nonzero band gap (*E*_*g*_) and a doable architecture realization into electronic heterostructures^33^,^34^. For example, single layer MoS_2_, an hexagonal two dimensional transition metal dichalcogenide (Fig. 1a,b)^3^,^9^,^29^,^30^,^35^, presents a large intrinsic band gap of 1.8 eV, and has been proposed as a perfect transistor^9^ with potential application in spintronic devices^28^,^29^,^36^,^37^.

Although in some cases theoretical studies of 2D materials had preceded their physical isolation^32^,^38^,^39^, recently the experimental results incentivize addressing the subject by different theoretical approaches. In particular, the use of Density Functional Theory (DFT) has promptly contributed with suitable results on the electronic, vibrational and optical properties of several 2D materials, in particular of 2D-MX_2_^11^,^12^,^40^.

The amount of DFT studies for 2D-MX_2_ in different configurations is vast, for example: studies of the physical properties of 2D-MX_2_ under dimensional confinement in the shape of ribbons^41^, the formation of 2D-MX_2_ heterostructures^13^, the effect of external electric fields^42^,^43^,^44^, the effect of defects in the morphology by atomic doping^45^, the alteration by chemical functionalization^46^,^47^ or the effect of applying mechanical strain^40^,^48^,^49^.

Experimentally, some studies report 2D-MX_2_ spintronics^27^,^29^,^50^,^51^,^52^,^53^,^54^,^55^,^56^,^57^,^58^,^59^, however only few computational works have explicitly considered the spin-orbit effect in selected compounds, *i.e.* MoS_2_, MoSe_2_, MoTe_2_, WS_2_, WSe_2_ and WTe_2_^30^,^49^,^54^,^59^,^60^,^61^,^62^,^63^,^64^.

Theoretically and with DFT, the use of fully relativistic pseudopotentials, rather than of scalar ones, accuratelly predict the dispersion of transition metals *d* orbitals^28^,^65^,^66^,^67^, thus the importance of their use to calculate the electronic properties of 2D-MX_2_. A remarkable feature predicted by considering the spin orbit effects in non-magnetic semiconductors 2D-MX_2_ is the splitting of their valence bands, which cannot be observed with the common scalar pseudopotential approximations^28^. Furthermore, experiments with MoS_2_, MoSe_2_, MoTe_2_, WS_2_, WSe_2_ and WTe_2_ confirm the “giant” spin orbit effects, supporting the use of fully relativistic pseudopotentials and positioning them as candidates for valleytronics^29^,^34^,^50^,^68^,^69^. Specifically, MoTe_2_ has been recently proposed as an outstanding material for excitonic devices^51^,^56^.

Therefore, the main motivation of the present work is to present the most commonly reported 2D-MX_2_ semiconductors^11^ in the frame of fully relativistic calculations, unveiling the effects of the spin-orbit interaction, specifically in the following 2D-compounds: ScO_2_, ScS_2_, ScSe_2_, CrO_2_, CrS_2_, CrSe_2_, CrTe_2_, MnO_2_, NiO_2_, NiS_2_, NiSe_2_, MoO_2_, MoS_2_, MoSe_2_, MoTe_2_, WO_2_, WS_2_ and WSe_2_. In this text, the electronic properties of the selected materials are presented, emphasizing the difference between calculations with the spin orbit interaction and without it, and comparing with the available experimental results. Within the results, separate sections are dedicated to the magnetic and nonmagnetic semiconductors. A criterion of the spin orbit effect is reported in terms of the shrinkage of the band gap and the splitting of the valence band maximum (VBM).

## Results

**Figure 1:**
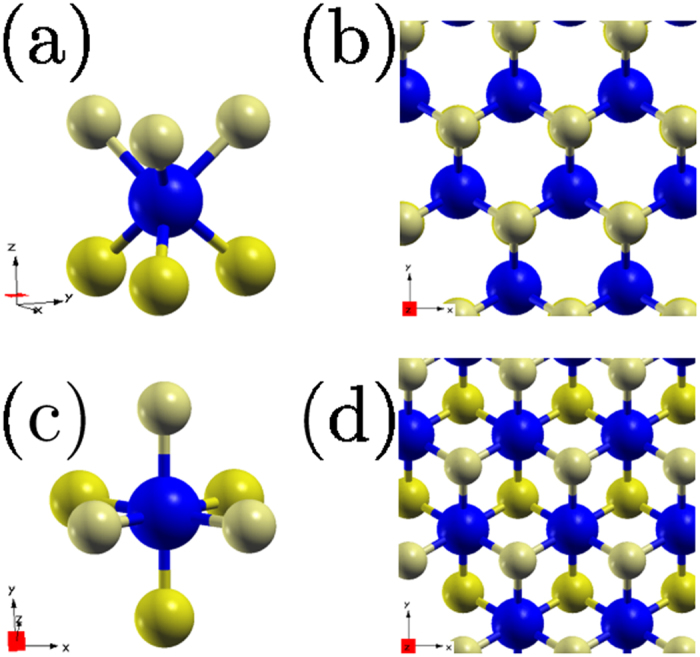
Schematic of the *H* and *T* structures of 2D-MX_2_ systems. (**a**) *H* structure in a trigonal prismatic perspective and (**b**) *xy* plane view of the *H* structure. (**c**,**d**) correspond to the *T* structure in the octahedron perspective and in the *xy* view respectively. Blue circles represent the layer of metallic atoms sandwiched between top (light yellow circles) and bottom (dark yellow circles) layers of dichalcogenide atoms.

Although 2D-MX_2_ compounds could exist either in the honeycomb (*H*, trigonal prismatic), centered honeycomb (*T*, octahedral) or distorted honeycomb (*T*′)^70^ structures, in this work we performed calculations focusing in the semiconducting 2D-MX_2_, specifically in their more energetically stable forms according to theoretical DFT calculations. Thus the majority of the structures were studied in the *H* configuration, with the exception of MnO_2_ and NiX_2_ studied in the *T* configuration (Fig. 1). Additionally, a special mention is done to metallic WTe_2_ in its most stable *T*′ structure, for its relevance^16^,^49^.

Starting with the lattice parameters and the electronic band gaps as obtained by SRUP (after the benchmark with Vanderbilt pseudopotential calculations, as explained in the methodology), we compare our generalized gradient approximation (GGA) results with those reported in ref. 11 obtained with DFT within the local density approximation (LDA). The lattice parameters from our calculations are shown in Table 1 (all atomic coordinates are available in Supplementary Information, SI); they turned to be roughly 2% larger than those reported in ref. 11, a consequence from a finer cutoff thresholds used in our calculations, and from the underestimation of the LDA approach. In contrast, our values strictly reproduce the results obtained with the GGA approximation by Rasmussen *et al.*^14^ and Zibouche *et al.*^64^. In 2D systems, the electronic properties are highly sensitive to minor changes in the lattice parameters^40^, thus the cohesive energies (*E*_*C*_) and *E*_*g*_s in our work are slightly different to those reported by Ataca *et al.*^11^ and Kang *et al.*^62^. However our *E*_*g*_s are very close to the scalar relativistic results by Zibouche *et al.*^64^.

The general features of all the band structures calculated with SRUP closely reproduce those reported in refs 11, 14, 64 and 71; specifically the existence of a band gap and the presence of magnetism, as shown in Table 1. The *E*_*C*_s relative to the free constituent atoms calculated with SRUP are also presented in Table 1. There, *E*_*C*_[*MX*_2_] = *E*_*T*_[*M*] + 2*E*_*T*_[*X*] − *E*_*T*_[*MX*_2_], where *E*_*T*_[*MX*_2_] is the total energy of the MX_2_ and *E*_*T*_[*M*] and *E*_*T*_[*X*] the total energies of the corresponding free M and X atoms. Although in general, the presented *E*_*C*_s are slightly larger than those reported by Ataca *et al.*^11^ (due to the use of finer force and energy cutoff thresholds in the present work), the trend is preserved, *i.e.* the highest cohesive energy belongs to MO_2_, and it decreases for MS_2_, MSe_2_, with the lowest value for MTe_2_. After full geometry optimization relaxations performed with SRUP, the final atomic positions and cell parameters were used as input in the FRUP geometry optimization calculations. Neither the atomic positions, nor the cell parameters differ between FRUP and SRUP optimizations (all geometry data is available in SI).

**Figure 2:**
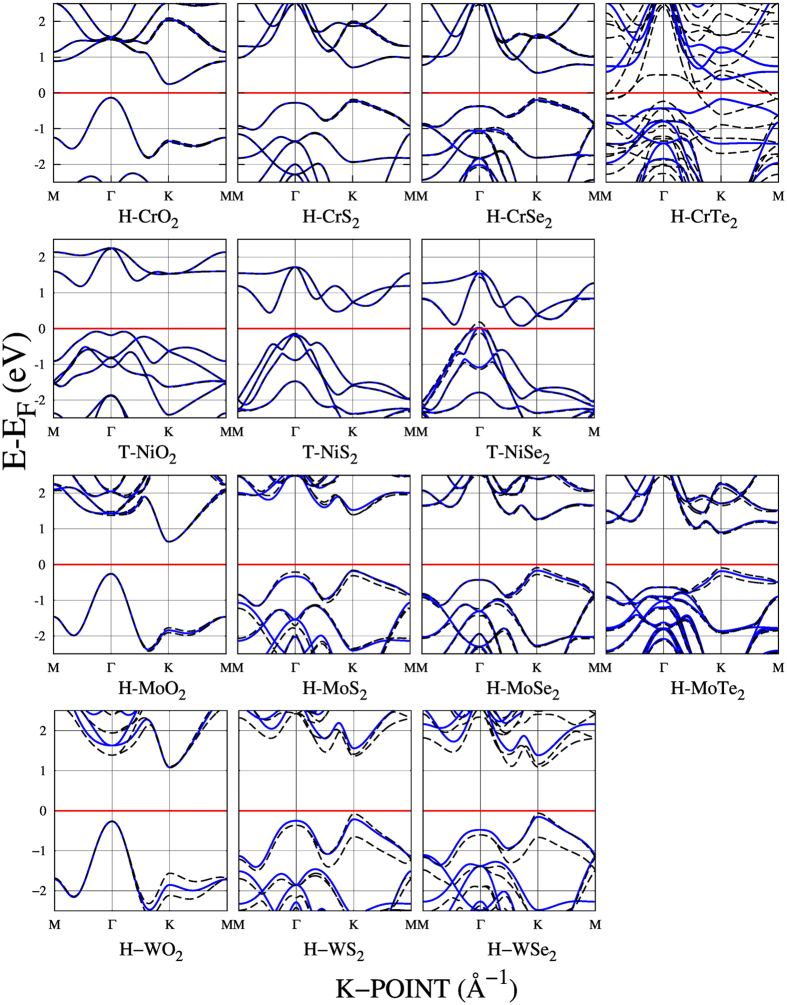
Electronic band structure of nonmagnetic 2D-MX_2_ semiconductors calculated with spin orbit interaction (dash-black) and without it (solid-blue). CrTe_2_ and NiSe_2_ turn from semiconductor to metal when calculated with FRUP, and CrTe_2_ turns magnetic.

Regarding the values of band gaps and magnetizations (*μ*), when the spin orbit is not taken into account (*i.e.* SRUP calculations), CrX_2_, NiX_2_, MoX_2_ and WX_2_ behave as nonmagnetic semiconductors, while MnO_2_ and ScX_2_ behave as magnetic, with *μ* values in good agreement with those reported in refs 11, 12, 14, 64 and 72 (see Table 1 and Fig. 2). Noncollinear calculations (*i.e.* FRUP calculations) are carried out in order to include the spin orbit effect. As a result, CrTe_2_ and NiSe_2_ present a change in their behavior from semiconductor to metal; moreover, CrTe_2_ turns from nonmagnetic to magnetic with a large *μ* of 1.59 *μ*_*B*_, in clear contrast to the *μ* = 0 reported by Ataca *et al.*^11^ and Rasmussen *et al.*^14^.

**Figure 3:**
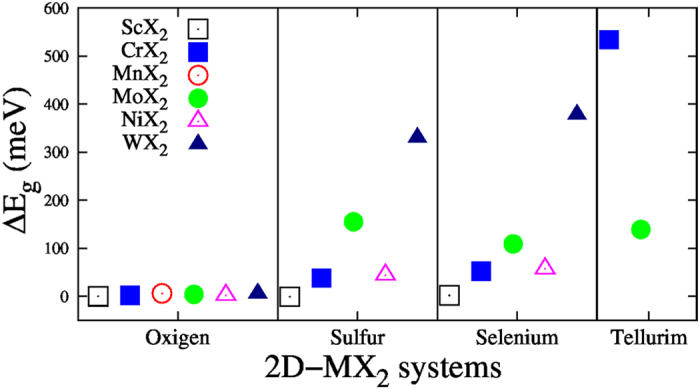
Difference between FRUP and SRUP band gaps (Δ*E*_*g*_). Transition metal dioxides Δ*E*_*g*_’s present the minimum FRUP alteration. Transition metal dichalcogenide Δ*E*_*g*_’s are in the range of ~10 to ~530 meV with CrTe_2_ presenting the largest effect with Δ*E*_*g*_ = 534 meV.

A clear effect of FRUP calculations is the splitting of originally spin degenerated bands as calculated with SRUP (Fig. 3 and Table 2). In particular, this effect in the bands close to the band gap is reflected in the difference between SRUP and FRUP band gaps, here reported as 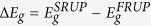.

FRUP results are presented in the following subsections. The compounds are categorized according to their magnetic behavior as obtained with SRUP calculations, and sub classified according to their most stable structure, either belonging to the *H* or *T* families, *i.e.*: CrX_2_, MoX_2_ and WX_2_ (X = O, S, Se and Te) belong to the nonmagnetic *H* family, NiX_2_ (X = O, S and Se) to the nonmagnetic *T* family, ScX_2_ (X = S, Se and Te) belong to the magnetic *H* structures and finally MnO_2_ is the only member of the magnetic *T* family.

### 0.1 Nonmagnetic

In this section the compounds that calculated with SRUP resulted nonmagnetic are analyzed; first we present MX_2_ structures with M = Cr, Mo and W, which are more stable in the *H* configuration, followed by NiX_2_ with the mos stable configuration in the *T* structure.

All MX_2_s with X = O present indirect band gaps as calculated with SRUP (continuum blue lines in Fig. 2), with their *E*_*g*_s values increasing as the atomic number grows, *i.e.* from a value of 0.381 eV for CrO_2_ to a value of 0.898 eV for MoO_2_ and finally a value of 1.349 eV for WO_2_. The VBMs are situated in the Γ points, whereas the conduction band minimums (CBM), are at the *K* points. In contrast, when X = S, Se and Te, (and M = Cr, Mo and W), the band gaps are direct, reducing as the atomic number grows (see band gap reduction from left to right in Fig. 2). For all the direct semiconducting *H* structures, both VBMs and CBMs are located at the *K* points.

### H structures

#### MO_2_

In general, MO_2_ structures present a small difference between bands calculated with FRUP and SRUP. Their Δ*E*_*g*_s are around a few meV (first column of Figs 2 and 3, and Table 2). However, the effect of the spin orbit inclusion is more noticeable around the *K* points, where locally flat VBMs calculated with SRUP split for FRUP calculations (*e.g.* the giant splitting = 556 meV for H-WO_2_ in Fig. 2 and Table 2). These locally flat bands -obtained with SRUP- result from an accumulation of *d* orbitals, emerging from the transition metals^73^ (see the density of states (DOS) in the SI Figs 1, 3 and 5, where narrow bands appear between −2.5 and −1.0 eV). In contrast, these bands disperse when the FRUP approximation is used^73^ (compare SI 1, 3 and 5 with SI 2, 4 and 6 respectively). Thus, it is required to consider the spin orbit interaction in order to obtain accurate electronic structures of systems involving transition metals^65^,^66^. Here WO_2_ is proposed as an experimental case of study in spintronic/valleytronics^29^,^62^.

#### CrX_2_ with X = S, Se and Te

**Figure 4:**
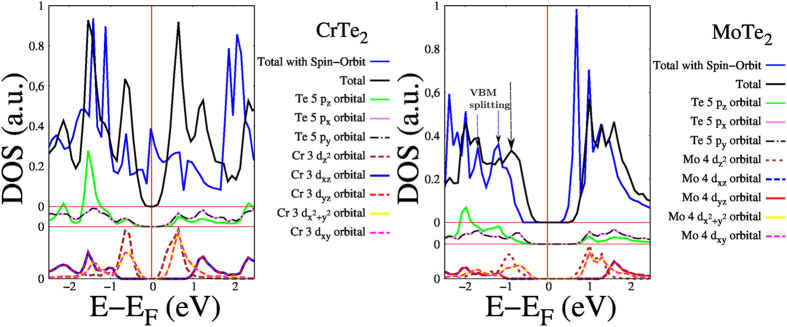
DOS and orbital-projected DOS for CrTe_2_, MoTe_2_ and WTe_2_. 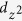
 (dash brown), 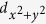 (solid yellow) and *d*_*xy*_ (dash pink) are the main SRUP orbitals contributors from the VBM to the DOS (solid black). The major effect of the spin orbit reflects in the dispersion of these orbitals (solid blue). *p* and *d* orbitals are shifted for clarity. The arrows indicate the splitting of the VBM for the FRUP calculation for MoTe_2_.

Bulk CrS, CrSe and CrTe compounds, in contrast with their corresponding 2D nonmagnetic semiconductor structures, present magnetism^74^. The calculations for 2D CrX_2_ systems with FRUP yield a small effect due to the spin orbit interaction. For CrS_2_ and CrSe_2_, they remain as semiconductors (Figs 2 and 3). Differently, for CrTe_2_ the inclusion of spin orbit interaction turns it from a nonmagnetic semiconductor with a band gap of 0.534 eV, to a magnetic metal with a *μ* of 1.59 *μ*_*B*_; in agreement with its metallic magnetic behavior reported by Lebegue *et al.*^12^. Looking at the local DOS (LDOS) in Fig. 4, it is clear that the principal contributions around the VBM and CBM, without spin-orbit effect, are mainly due to the contribution of the Cr 3*d* and Te 5*p* orbitals; specifically the 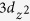, 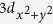 and 3*d*_*xy*_ orbitals that disperse in some degree when the fully relativistic approximation is considered^60^ (Figs 2 and 4). CrTe_2_ presents a Δ*E*_*g*_ = 534 meV, the largest among all the systems reported in this work. CrS_2_ and CrSe_2_ present small VBM splittings, at the *K* points, of 69 and 95 meV respectively. In contrast CrTe_2_ presents a giant splitting of 2.32 eV, with the already mentioned magnetic moment of 1.59 *μ*_*B*_.

For the sake of completeness, we looked into the experimentally reported structures at the Inorganic Crystal Structure Database (ICSD), finding the existence of CrS_2_ (ICSD 75420)^75^, CrSe_2_ (ICSD 626718)^76^ and TlCrTe_2_ (ICSD 152836)^77^ as layered bulk materials, either in the *T* or *T*′ forms. From these information, 2D structures of CrS_2_, CrSe_2_ and CrTe_2_ were built in the *T*′, *T* and *T* forms respectively. The structures were geometry optimized and their electronic properties calculated (see band structures and DOSs in the SI Figs 16–21). For both approximations, SURP and FRUP the systems in the *T* or *T*′ structures are metallic. Regarding the cohesive energies, the structures in the *T* or *T*′ forms are ~1.65, 0.54, and 0.76 eV less stable than the above mentioned semiconducting *H* structures, respectively. The FRUP results for CrS_2_, CrSe_2_ and CrTe_2_ in the *T* and *T*′ structures present magnetism, with *μ* ~ 1.09, 0.08 and 0.33 *μ*_*B*_ respectively, in contrast with the non magnetic *H* CrS_2_ and CrSe_2_ semiconductors, and with the magnetic metal *H* CrTe_2_ with *μ* = 1.59 *μ*_*B*_.

#### MoX_2_ with X = S, Se and Te

Now we present the results for some of the most studied metal-dichalcogenide monolayers; mainly MoS_2_ and MoSe_2_^3^,^6^,^7^,^8^,^9^,^10^,^11^,^12^,^13^,^29^,^34^,^50^,^64^ and the emerging MoTe_2_^51^,^55^,^56^,^64^. The effect of the spin orbit inclusion in their band gaps is shown in Fig. 3. For this group, the largest Δ*E*_*g*_ is for MoS_2_ with a value of 155 meV. Experimentally available *E*_*g*_s shown in Table 2 present a fair agreement with the calculated values.

Considering the effect around the *K* point, the trend is the same as for Cr and W (Table 2); the VBMs split with the bands separation growing from S to Te as the atomic number increases. As mentioned before, with FRUP, the DOSs of the valence bands are disperse near the Fermi Energy (*E*_*F*_), in contrast to some narrow peaks present in DOSs obtained with SRUP (SI, Figs 3 and 4).

Theoretically, the splittings at the *K* point for MoS_2_, MoSe_2_ and MoTe_2_ are 151, 188 and 219 meV respectively (Table 2), data in a good agreement with refs 60, 61, 62 and 64, providing extra benchmarks for the used FRUP pseudopotentials. Experimentally, the energy differences between A and B excitons, attributed to the spin-orbit induced valence band splitting, measured via photoluminescence (PL) has been reported for MoS_2_, MoSe_2_ and MoTe_2_ as shown in Table 2 35,51,53,55,56,59,78,79. Calculated splittings are close to the experimental values attributed to the exciton binding energy in the case of MoS_2_ and MoSe_2_. For MoTe_2_, the calculated value of 219 meV is close to two of the experimentally reported values, and in contrast with a higher value of 580 meV in ref. 51.

The orbital-projected DOS for MoTe_2_ in Fig. 4 is presented in order to show the accumulation of Mo 4*d* orbitals close to the *E*_*F*_. Specifically the 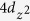, 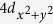 and 4*d*_*xy*_ ones are the closest to the *E*_*F*_, and therefore, the orbitals where the splittings are expected to occur and actually occur, as shown in the MoTe_2_ panel of Fig. 2, where the VBM splits in two bands^32^,^73^ (Table 2).

The giant spin-orbit-induced spin splitting supports the proposal of MoS_2_, MoSe_2_ and MoTe_2_ as candidates for their experimental valleytronics studies^29^,^30^,^31^.

#### WX_2_ with X = S and Se

Finalizing the nonmagnetic *H* compounds, the group of WX_2_ is analyzed. As in the previous cases, there is a growing tendency in Δ*E*_*g*_ as the atomic number of the chalcogenide species increases (Fig. 3). The experimental and theoretical values of *E*_*g*_s are close.

Regarding the VBM splittings, the values are reported at the *K* points in Table 2. As for MoS_2_ and MoSe_2_, the results for WS_2_ and WSe_2_ are in agreement with those reported in refs 60, 61, 62 and 64, in both terms, of band gap and VBM splitting. Experimental value, from the energy difference between the A and B excitons for WS_2_ is reported in Table 2 in good agreement with the calculated one. Here WO_2_ is proposed as an experimental case of study in spintronic/valleytronics 29,62.

Since some theoretical studies report WTe_2_ system as a semiconductor in the H structure^11^,^62^,^64^, here we calculate it finding that the H structure is a metastable one with a bandgap of 1.060(0.649) eV for SRUP(FRUP) (See band structure and DOS in Fig. 15 of SI). This structure present a giant Δ*E*_*g*_ of 411 eV and a VBM splitting at the *K* point of 609 meV. However, we also looked into the experimentally reported structures at the ICSD, finding the existence of WTe_2_ (ICSD 73323)^80^ as layered bulk material in the *T*′ form. We built the *T*′ structure, optimized it and found it ~0.03 eV more stable in terms of cohesive energy with respect to the meta stable *H* structure. Interestingly, the *T*′ WTe_2_ system is metallic from SRUP and FRUP calculations (see band structure and DOS in Figs 22 and 23 of SI), in agreement with the experimental and theoretical data in refs 16, 27 and 49.

### T structure

#### NiX_2_ with X = O, S and Se

NiX_2_ systems are the only compounds in the energetically more stable *T* configuration of the nonmagnetic group (Fig. 1c,d). The VBM of NiO_2_ presents a bimodal behavior around the Γ point, whereas the CBM is located at 3/4 of the *M* − Γ path. In the case of NiS_2_ and NiSe_2_ the VBMs are closer to the Γ point and the CBMs locate in the Γ − *K* path, almost at the *K* point. All the band gaps for Ni systems are indirect.

Figure 2 shows that NiO_2_ and NiS_2_ are indirect semiconductor for both, SRUP and FRUP calculations. The spin orbit inclusion produces negligible alterations at their band structures with VBM splittings ~1 meV. Within this group, the case of interest is NiSe_2_, indirect semiconductor for SRUP and metallic when the spin orbit effect is considered^41^ (Fig. 2). A close up to the two highest SRUP valence bands (SI Fig. 9) reveals a degeneration for the VBM exactly at the Γ point. In contrast, FRUP brakes the degeneration with a giant band splitting of 302 meV, turning metallic as a result of the Fermi level crossing by the VBM.

### 0.2 Magnetic

**Figure 5:**
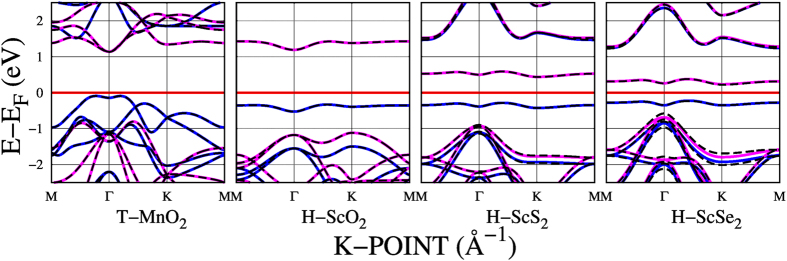
Electronic band structure of magnetic 2D-MX_2_ semiconductors. SRUP bands are represented in solid blue (spin up) and solid magenta (spin down) lines. FRUP bands are plotted in dashed black lines.

In this last section we present all the compounds that calculated with SRUP are magnetic semiconductors in agreement with ref. 14, specifically T-MnO_2_, H-ScO_2_, H-ScS_2_ and H-ScSe_2_ (Fig. 5). After the inclusion of the spin orbit interaction the magnetic and semiconductor characters remain, with magnetization(band gap) of 2.98(1.23), 1.0(1.52), 0.97(0.72) and 0.84(0.45) *μ*_*B*_(eV) respectively. To explore the effect of spin orbit, the focus is on the regions of the band structure where spin degeneration appears, as previously done for the NiSe_2_ band degeneration.

#### ScX_2_ with X = O, S and Se

This group presents the *H* structure as the energetically more stable configurations (Fig. 5 and Table 1). The VBMs and CBMs calculated with FRUP and SRUP coincide; however, the ante-penultimate and penultimate bands present some differences between FRUP and SRUP calculations. Zooming into the band structures allows to appreciate the effect of the inclusion of spin orbit (SI Fig. 14), with splitting of the referred bands ~10, 30 and 100 meV at the Γ point for ScO_2_, ScS_2_ and ScSe_2_ respectively. Furthermore, the SRUP (FRUP) energy difference between the ante-penultimate and the penultimate bands at the *K* point are 160(209) and 133(322) meV for ScS_2_ and ScSe_2_, respectively, *i.e.* band splittings ~50 and 190 meV. *H* ScO_2_ presents an *E*_*g*_ and a *μ* of 1.521 eV and 1*μ*_*B*_ respectively, in agreement with ref. 11 and in contrast with the work by Loh *et al.*^81^ reporting an antiferromagnetic metal behavior. Regarding ScS_2_, Zhang *et al.* have reported an *E*_*g*_ of 0.74 eV and a *μ* of 1 *μ*_*B*_^71^, in perfect agreement with our SRUP calculations.

#### MnO_2_

A magnetic semiconductor with *T* structure, is the last compound presented in this work: MnO_2_, with an *E*_*g*_ of 1.23 eV and a *μ* = 2.98 *μ*_*B*_, in good agreement with refs 14, 72 and 82 respectively (Fig. 5 and Table 1).

The inclusion of spin orbit for this compound is reflected at the bands below the VBM, specifically in the region close to the Γ point (SI, Fig. 11, from −1.4 to −1.0 eV). The band splitting with FRUP is 30 meV and its *μ* is not altered.

## Discussion

With a benchmarking purpose, the present work addresses by means of fully relativistic DFT calculations, the effect of the spin orbit interaction in a thorough study of the electronic and spin properties for several semiconductor monolayer transition metal dioxide and dichalcogenides.

MoS_2_, MoSe_2_, MoTe_2_, WS_2_, WSe_2_ and WTe_2_ exist as van der Waals solids (their crystalline structure is presented in SI) and have been the focus of several experimental and theoretical works. In this paper the developed fully relativistic potentials were benchmarked by comparison with previous theoretical results, and more importantly, by comparing the VBM spin splitting with the experimentally available results from PL, confirming the validity of the potentials derived in this work.

Although the focus has been on the previous mentioned materials, we have also turn our attention towards those materials that could exist in 3D and can be exfoliated into 2D materials.

In particular, regarding the MO_2_ family, they exist in the rutile-like form and have not been yet experimentally reported, neither in the *H* or *T* forms. Therefore, our results for M = Sc, Cr, Mn, Ni, Mo and W in MO_2_ systems, were only compared with theoretical works, presenting, in general, a good agreement, unless the case of ScO_2_, which we found as a magnetic semiconductor in agreement with ref. 11 but in contrast with the metallic behavior reported in ref. 81.

The spin-orbit effects are presented with a growing tendency as the atomic number increases (Table 2). The general feature in the block of dioxide transition metal monolayers is an indirect band gap (from Γ to *K*), with orbitals around *E*_*F*_ mainly due to presence of oxide *p*-orbitals. The metal *d*-obitals are not present around *E*_*F*_, and even that the valence bands split around the *K*-point, do not affect the values of the *E*_*g*_s. Motivating a future systematic study on the effects of spin-orbits in the rutile-like systems.

The giant spin-orbit effects found in several nonmagnetic semiconductors, supports the proposal of new materials as promising candidates for technological applications in valleytronics and spintronics (*e.g.* MoO_2_, MoTe_2_, NiSe_2_, WS_2_, WSe_2_ and WTe_2_)^28^,^29^,^30^,^60^,^83^. CrTe_2_ was found to behave as magnetic metal (with *μ* ~ 1.59 *μ*_*B*_) when using FRUP versus its semiconductor behavior when calculated with SRUP.

Within the magnetic semiconductors FRUP calculations, for metallic dioxides, the results almost coincide with the calculations not considering the spin orbit effect.

Finally, metallic dichalcogenide magnetic semiconductors are not affected in their main features, only at their inner valence bands, specifically in the regions where SRUP calculations present points of degeneration, appearing shifts and splits of bands.

This work confirms the requirement of using a fully relativistic pseudopotential approximation in order to accurately predict properties in most of the monolayers involving transition metals.

## Methods

*Ab-initio* calculations were performed with the Quantum ESPRESSO (QE)^84^ plane wave DFT and Density Functional Perturbation Theory code, available under the GNU Public License^85^. Spin polarized scalar relativistic calculations were performed for all the systems, and, in order to include the spin-orbit interaction, fully relativistic approximation was adopted^65^,^66^.

With the aim of using suitable pseudopotentials for transition metals, and generated by the same generation scheme, RRKJ pseudopotentials were chosen^86^. Scalar relativistic ultrasoft pseudopotentials (SRUP) and fully relativistic ultrasoft pseudopotentials (FRUP) for Mo, Ni, Se, S, O and Te, were accessible in the QE website repository^84^ within the RRKJ scheme. Meanwhile the pseudopotentials for Sc, Cr, Mn and W were not available and were generated through the ld1.x code, as implemented in QE^84^.

In order to benchmark the generated RRKJ pseudopotentials, the systems (MX_2_ with M = Sc, Cr, Mn and W, and X = O, S, Se and Te) were constructed and their lattice parameters and band structure calculated. Lattice parameters and band structures of all these structures were also calculated with the available pseudopotentials in the QE repository, specifically using PBE in the Vanderbilt scheme. The results from the RRKJ and the Vanderbilt pseudopotnentials were compared with perfect matching. Fully relativistic ultras of pseudopotentials (FRUP) were then built.

For the exchange-correlation, we used the Perdew-Burke-Ernzerhof (PBE) and GGA^87^,^88^. For the plane-wave basis sets, in all cases, we used converged energy cutoffs higher than 612 eV. The convergence energy parameter between consecutive self consistent field calculations was chosen as 10^−7^ eV. The maximum force acting on converged structures was smaller than 0.003 eV/Å, and the stress in the periodic direction was lower than 0.001 GPa. For the Brillouin-Zone integrations, Monkhorst-Pack grids^89^ of 16 × 16 × 1 *k*-points were used. The starting magnetization was randomly set to 1/2 *μ*_*B*_ on the metallic atoms.

Geometry optimization was performed using the conjugate gradient method, and the relaxed atomic positions and lattice parameters in the *xy* plane were obtained. For all *H* structures the relaxation was performed with symmetry constrains, providing the energetic minimum of the system. The size of the supercell in the *z* direction was fixed to 10 Å, providing enough distance to simulate 2D crystals, assuring isolation with the parallel adjacent supercell images.

## Additional Information

**How to cite this article**: Reyes-Retana, J. A. and Cervantes-Sodi, F. Spin-orbital effects in metal-dichalcogenide semiconducting monolayers. *Sci. Rep.* **6**, 24093; doi: 10.1038/srep24093 (2016).

## Supplementary Material

Supplementary Information

## Figures and Tables

**Table 1:** Structure, electronic and magnetic properties of MX_2_s.

MX_2_	Monolayerstructure	a (Å)	E_*C*_ (eV)	E_*g*_ (eV)		*μ* (*μ*_*B*_)		ExperimentalE_*g*_(eV)
				SRUP	FRUP	SRUP	FRUP	
ScO_2_	*H*	3.22	21.43	1.521	1.521	1.00	1.00	–
ScS_2_	*H*	3.79	16.18	0.721	0.722	0.97	0.97	–
ScSe_2_	*H*	3.95	14.29	0.456	0.454	0.84	0.82	–
CrO_2_	*H*	2.63	24.48	0.381	0.379	NM	NM	–
CrS_2_	*H*	3.05	19.39	0.929	0.891	NM	NM	–
CrSe_2_	*H*	3.22	17.15	0.756	0.704	NM	NM	–
**CrTe_2_**	*H*	3.48	14.57	0.534	**Metal**	NM	1.59	–
MnO_2_	*T*	2.96	24.00	1.230	1.224	2.98	2.97	–
NiO_2_	*T*	2.86	19.04	1.265	1.264	NM	NM	–
NiS_2_	*T*	3.33	15.64	0.561	0.517	NM	NM	–
**NiSe_2_**	*T*	3.51	14.04	0.094	**Metal**	NM	NM	–
MoO_2_	*H*	2.84	25.77	0.898	0.894	NM	NM	–
MoS_2_	*H*	3.20	21.11	1.706	1.551	NM	NM	1.90^78^, 1.89^53^
MoSe_2_	*H*	3.33	18.87	1.438	1.331	NM	NM	1.55^54^
MoTe_2_	*H*	3.55	16.36	1.116	0.979	NM	NM	1.10^55^, 1.08^56^
WO_2_	*H*	2.83	25.30	1.349	1.340	NM	NM	–
WS_2_	*H*	3.19	20.23	1.771	1.440	NM	NM	1.9^57^, 2.0^58^,^59^
WSe_2_	*H*	3.33	17.76	1.535	1.159	NM	NM	1.65^59^, FET^17^

For every compound the table includes: the energetically more stable configuration (trigonal prismatic -*H*- or octahedral -*T*-), lattice parameter (*a*), cohesive energy (*E*_*C*_), energy band gap (*E*_*g*_), and magnetization (*μ*). The SRUP columns correspond to the *E*_*g*_ and *μ* when the spin orbit interaction is not included. *E*_*g*_s and *μ*s are calculated and reported using the spin orbit inclusion (FRUP calculations). Materials that behave as semiconductors with SRUP and turn to metals with FRUP are bolded. Available references to the experimental *E*_*g*_s are included in the last column.

**Table 2:** Spin splitting effect at the *K*–point for nonmagnetic *H* structures.

MX_2_	Splitting (meV)	Experimental (meV)
CrO_2_	66	–
CrS_2_	69	–
CrSe_2_	95	–
CrTe_2_	2320	–
MoO_2_	138	–
MoS_2_	151	150^78^, 140^53^ 130^35^,^59^
MoSe_2_	188	180^79^, 210^59^
MoTe_2_	219	250^55^, 300^56^, 580^51^
WO_2_	556	–
WS_2_	571	400^59^, 410^58^
WSe_2_	603	450^59^

The effect is shown for the VBM of CrX_2_, MoX_2_ and WX_2_. The last column presents the available experimental energy differences between A and B exitons with their corresponding references.
