# Supplementary material for: Spin-orbital effects in metal-dichalcogenide semiconducting monolayers
Source: Sci Rep. 2016 Apr 20;6:24093. doi: 10.1038/srep24093 (PMC4837337; doi:10.1038/srep24093)
Supplement: Supplementary Information [file srep24093-s1.pdf]

Supplementary Information for: Spin-orbital effects in metal-dichalcogenide semiconducting monolayers

J. A. Reyes-Retana[1,*] and F. Cervantes-Sodi[1]

[1]Universidad Iberoamericana, Departamento de Física y Matemáticas, Prolongación Paseo de la Reforma 880, Lomas de Santa Fe,DF, 01219, México
[*]angelreyes@qe-ibero.com

ABSTRACT

Metal-dioxide & metal-dichalcogenide monolayers are studied by means of Density Functional Theory. For an accurate reproduction of the electronic structure of transition metal systems, the spin orbit interaction is considered by using fully relativistic pseudopotentials (FRUP). The electronic and spin properties of MX_2 (M=Sc, Cr, Mn, Ni, Mo & W and X=O, S, Se & Te) were obtained with FRUP, compared with the scalar relativistic pseudopotentials (SRUP) and with the available experimental results. Among the differences between FRUP and SRUP calculations are giant splittings of the valence band, substantial band gap reduction and semiconductor to metal or non-magnetic to magnetic "transitions". MoO_2, MoS_2, $MoSe_2$, $MoTe_2$, WO_2, WS_2 and WSe_2 are proposed as candidates for spintronics, while $CrTe_2$, with $\mu \sim 1.59\mu_B$, is a magnetic metal to be experimentally explored.

Band Structures and Density of States

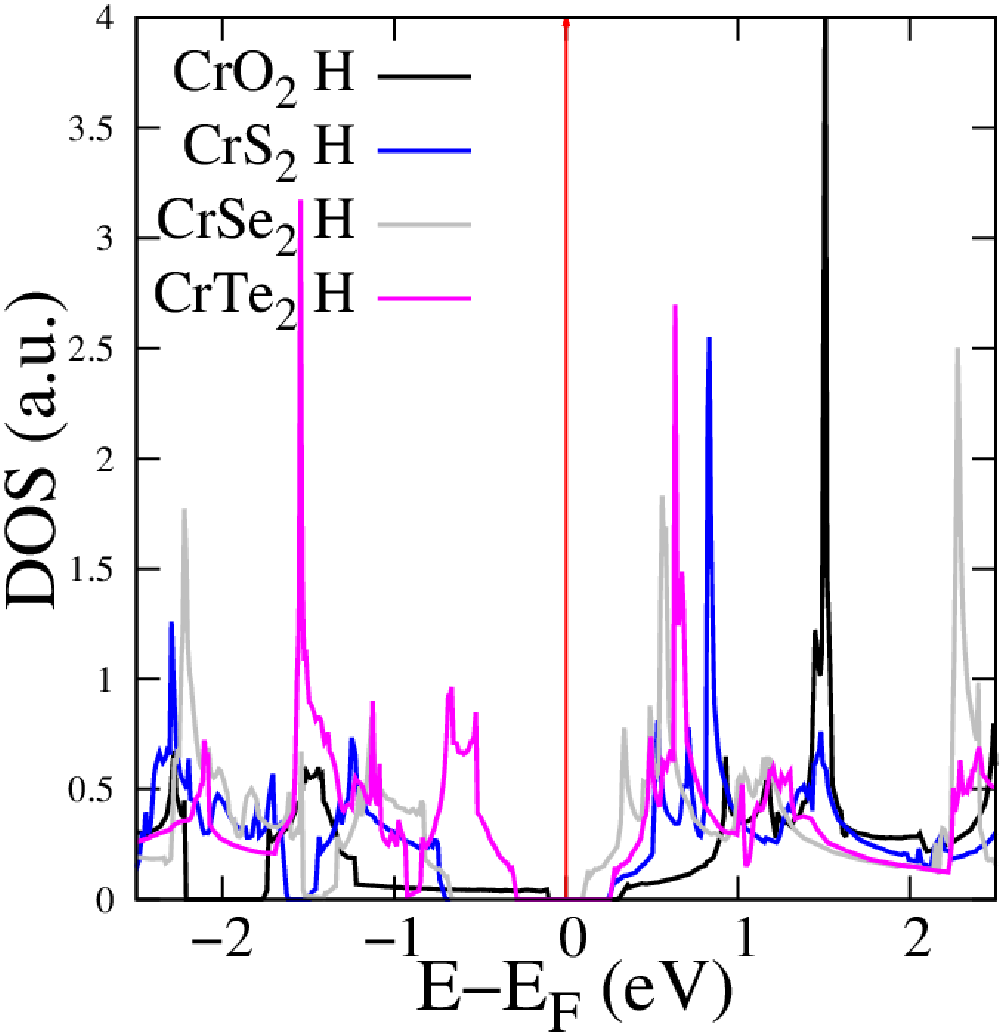

Supplementary 1. DOS of CrX_2, where X= O, S, Se and Te, as obtained with SRUP.

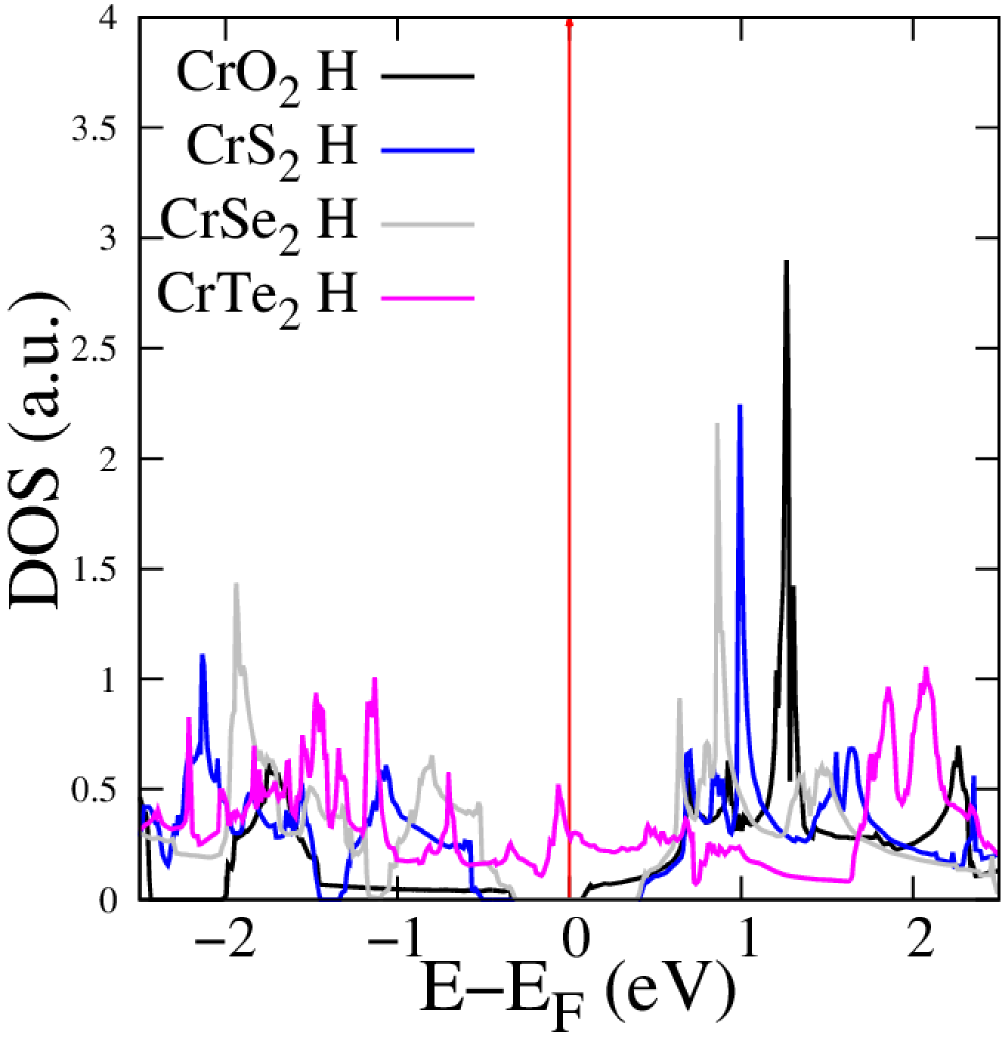

Supplementary 2. DOS of CrX_2, where X= O, S, Se and Te, as obtained with FRUP.

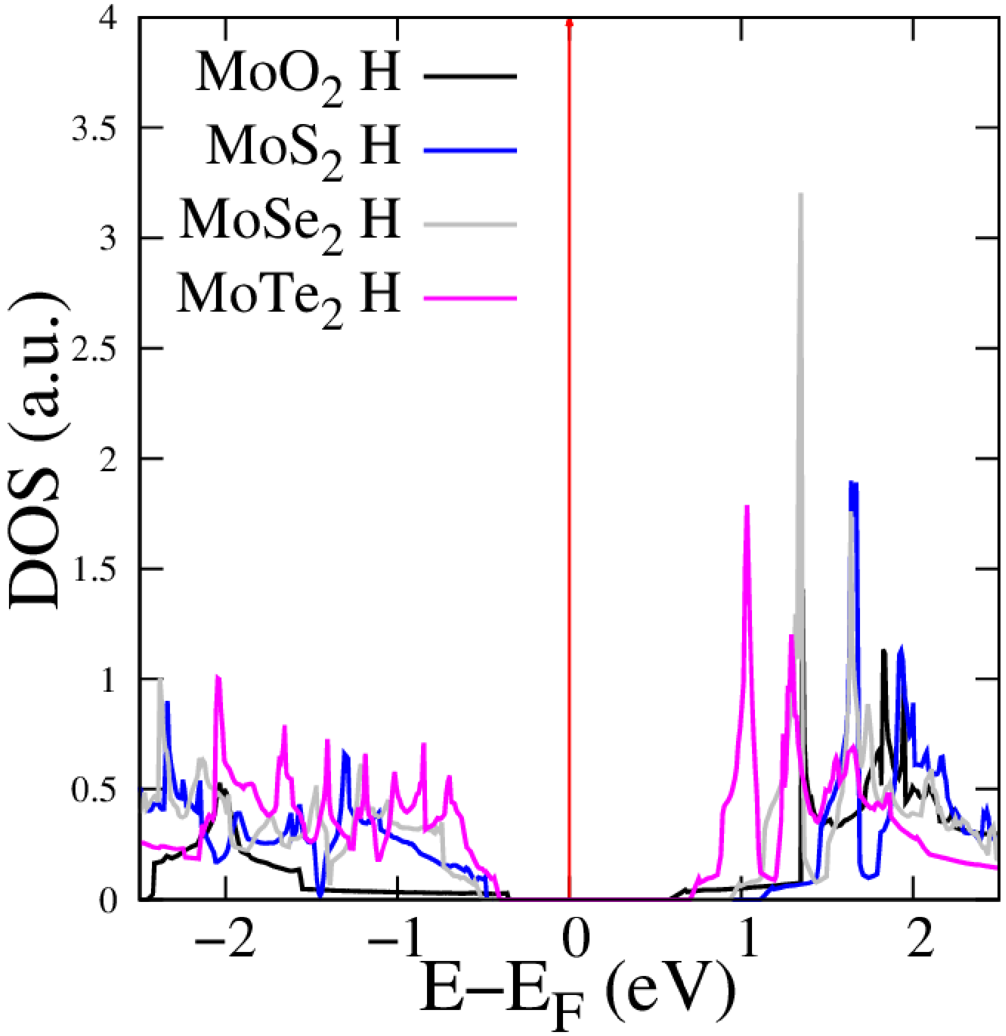

Supplementary 3. DOS of MoX_2, where X= O, S, Se and Te, as obtained with SRUP.

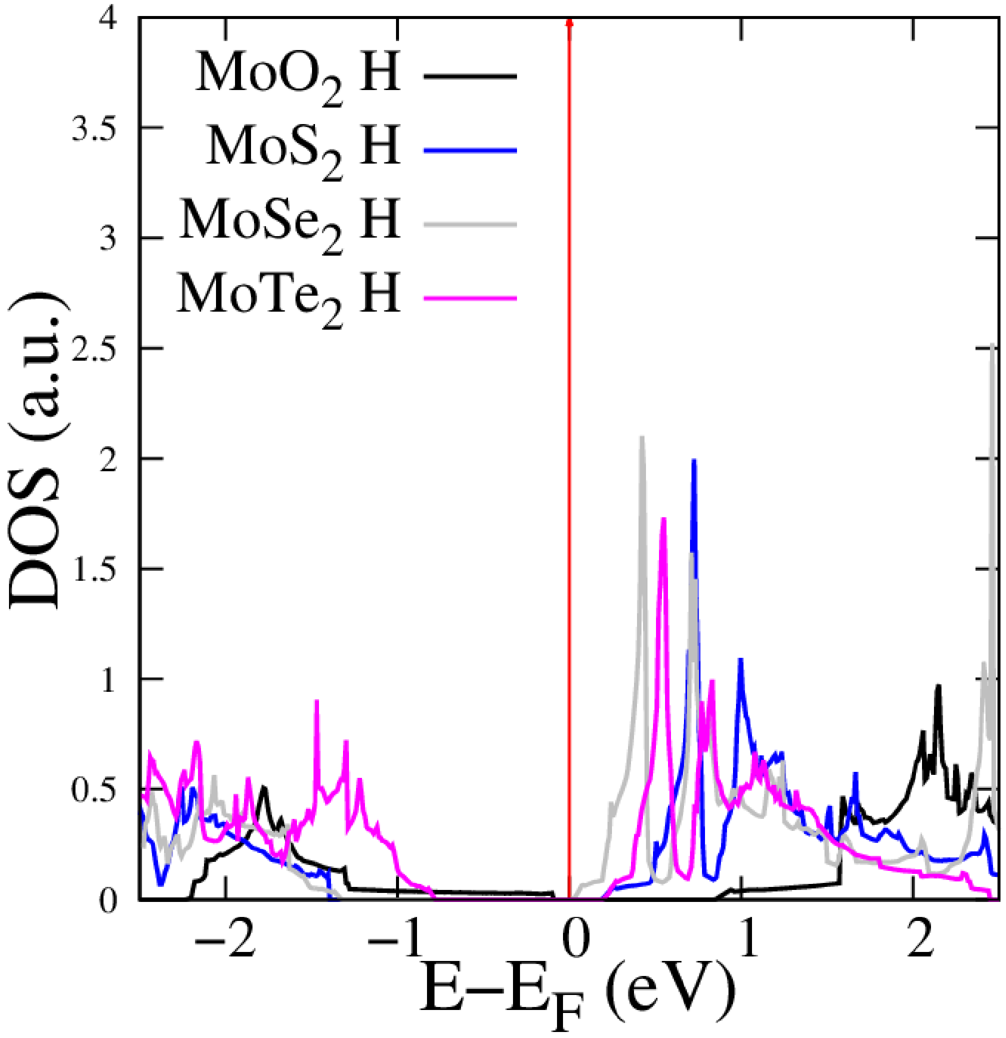

Supplementary 4. DOS of MoX_2, where X= O, S, Se and Te, as obtained with FRUP.

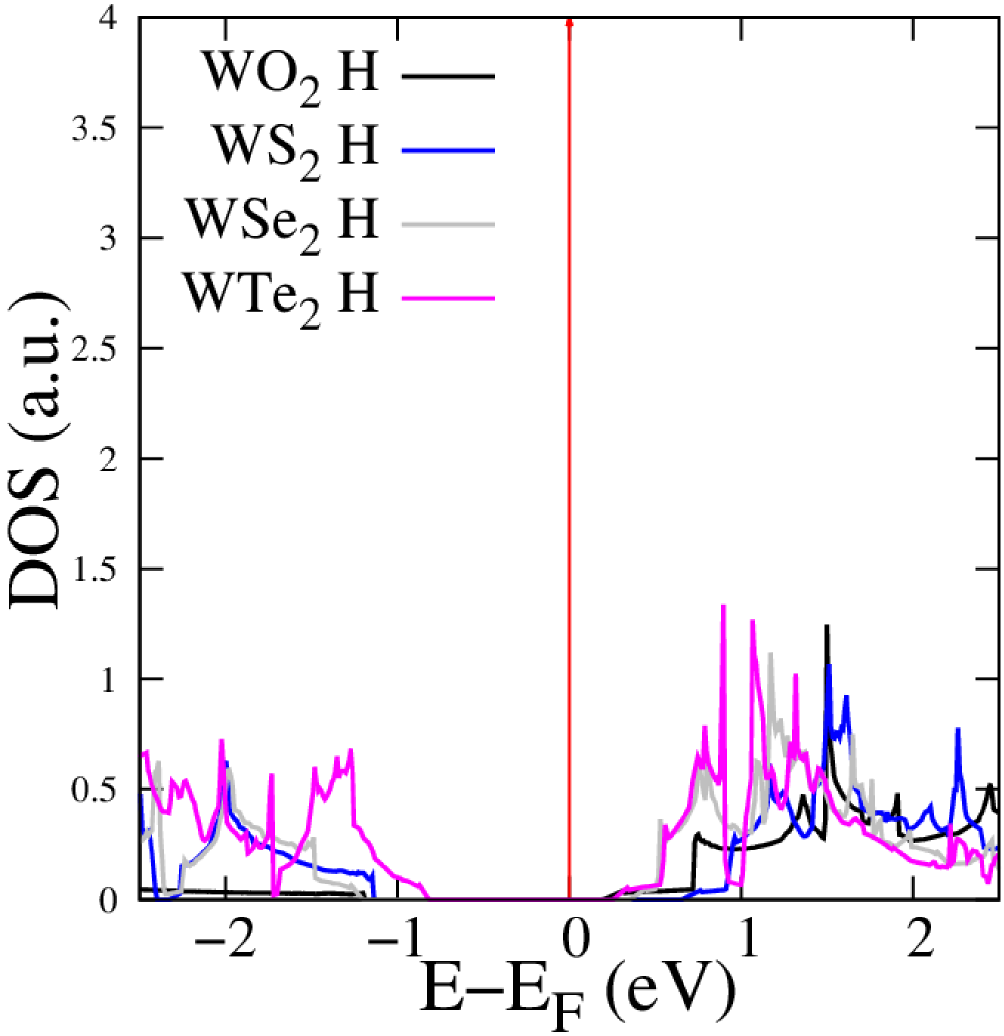

Supplementary 5. DOS of WX_2, where X= O, S, Se and Te, as obtained with SRUP.

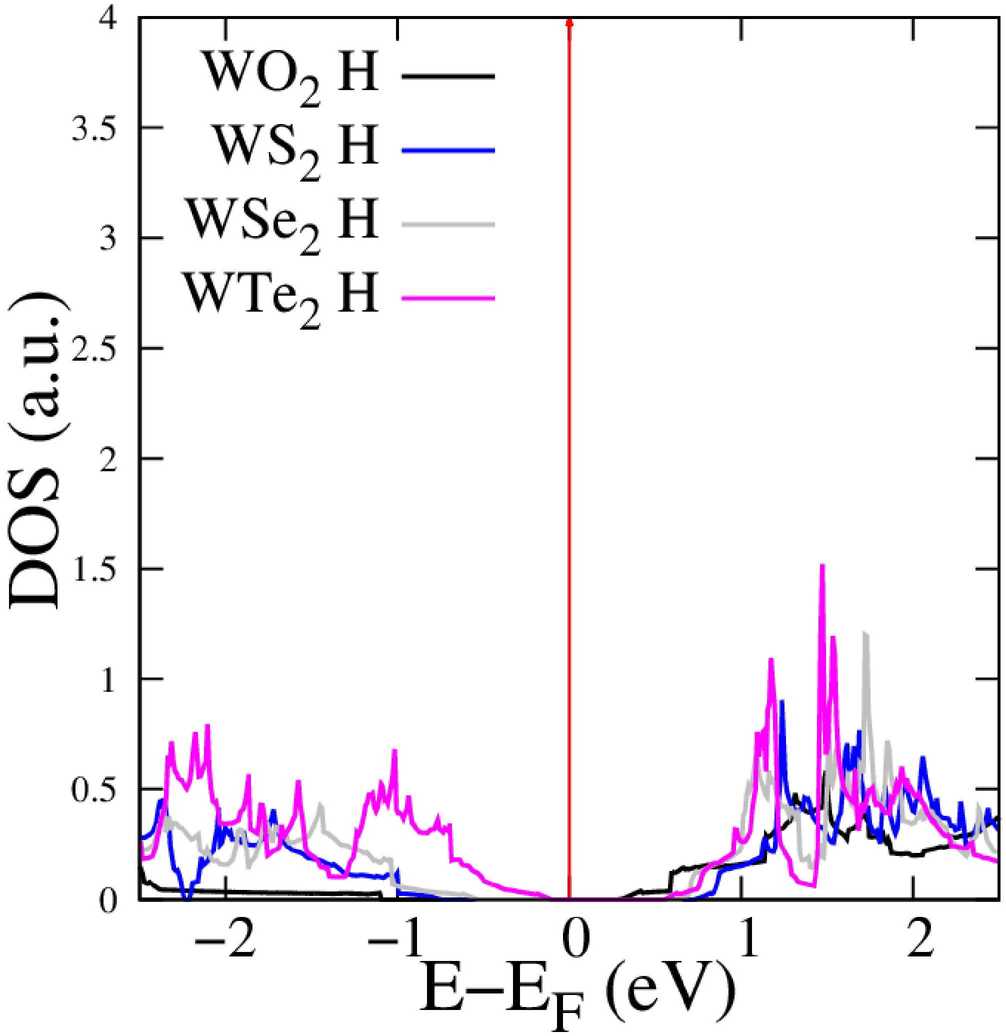

Supplementary 6. DOS of WX_2, where X= O, S, Se and Te, as obtained with FRUP.

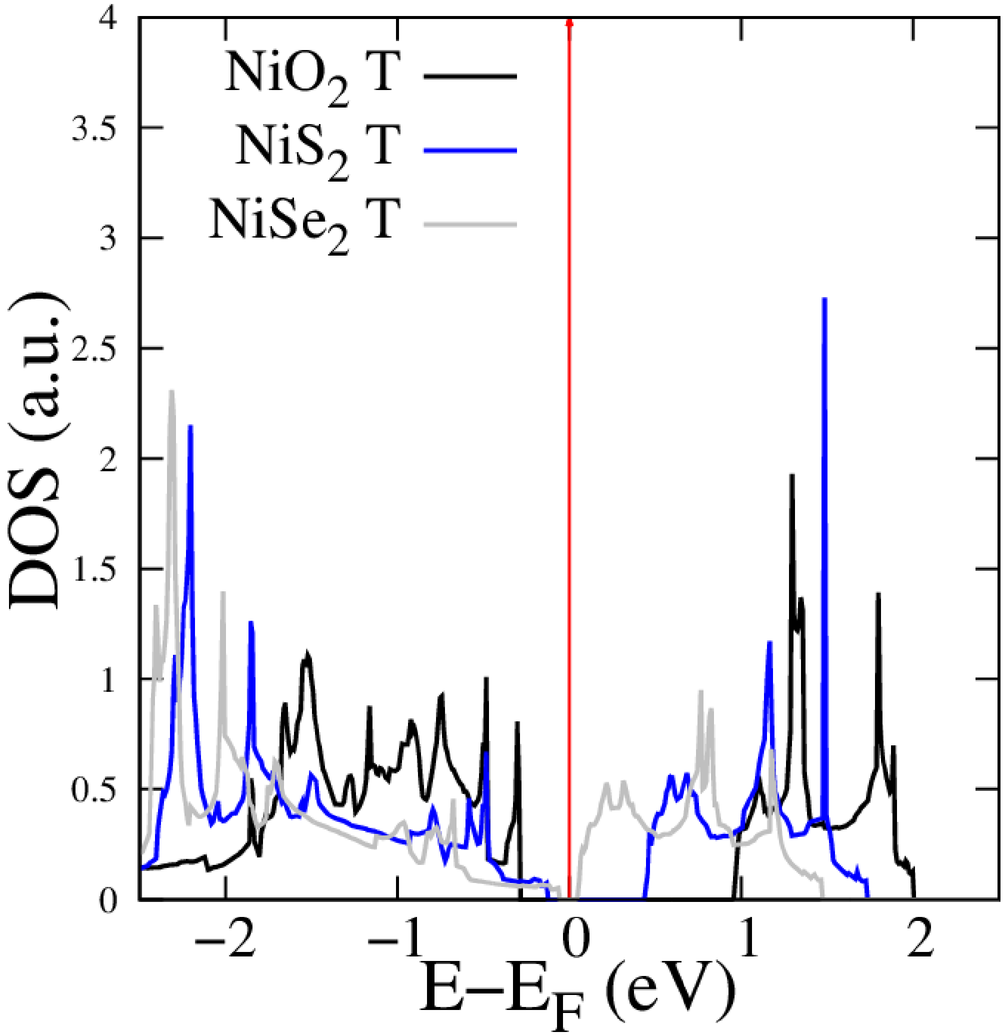

Supplementary 7. DOS of NiX_2, where X= O, S, Se and Te, as obtained with SRUP.

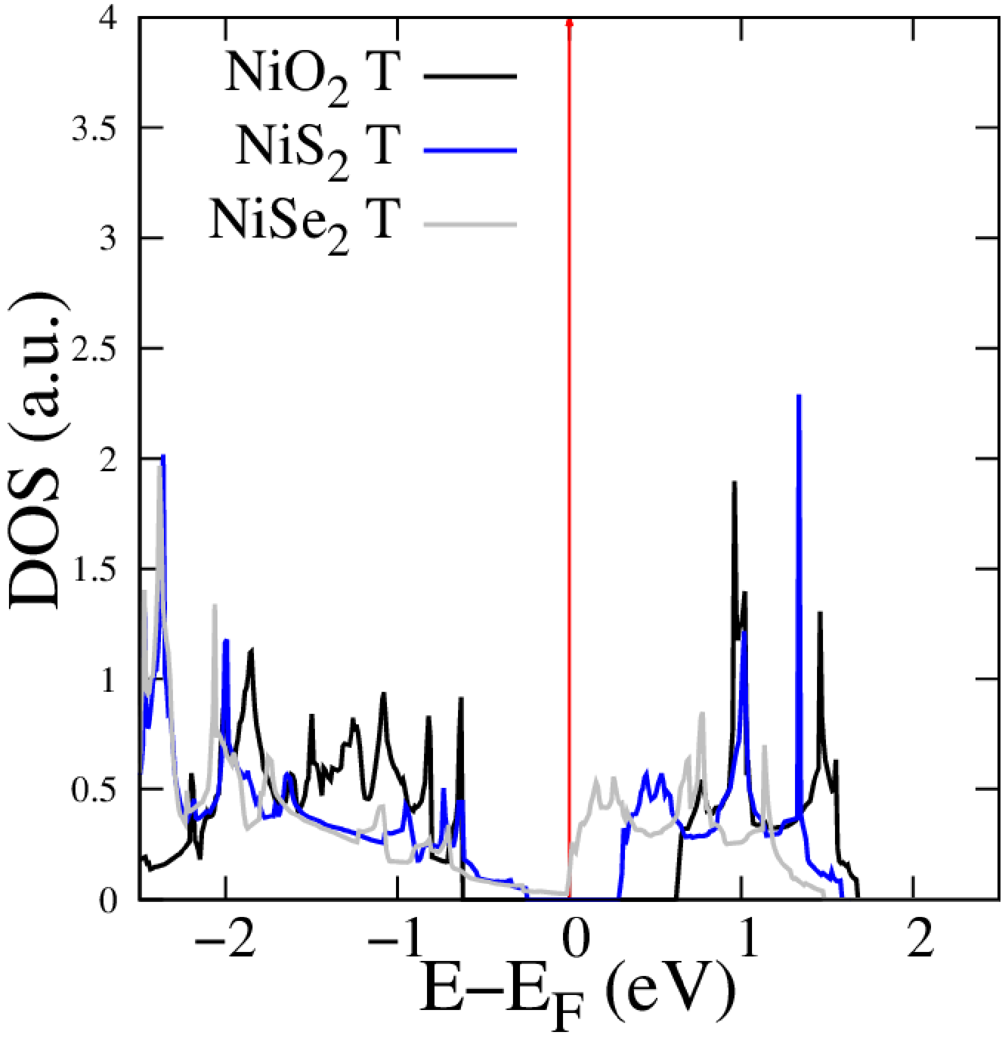

Supplementary 8. DOS of NiX_2, where X= O, S, Se and Te, as obtained with FRUP.

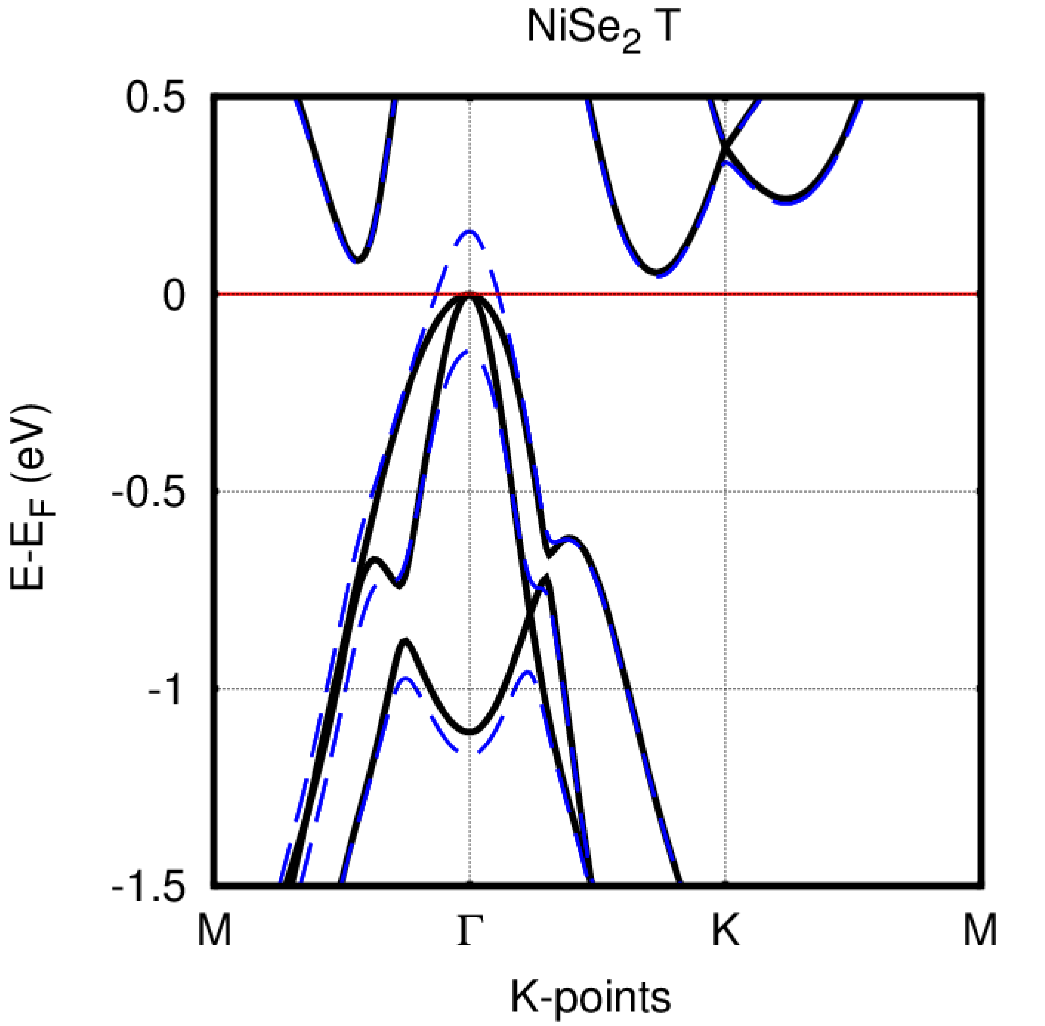

Supplementary 9. Zoom of electronic band structure for $NiSe_2$, showing the splitting of valance bands at the Γ point. Solid black and dashed blue lines represent degenerated spins and non collinear spins, respectively. The last two valance bands (dashed blue) present a separation of 302 meV, in comparison with the same bands (solid black) when FRUP calculations are considered.

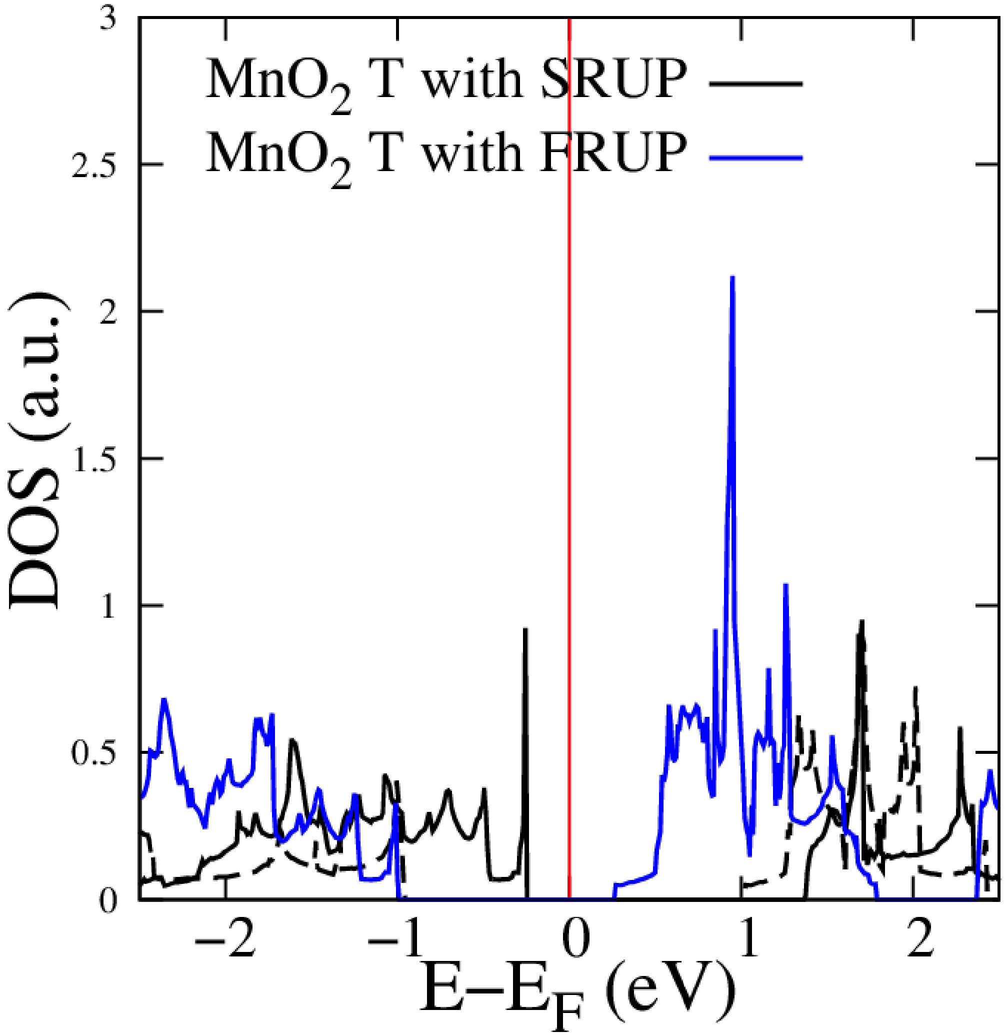

Supplementary 10. DOS of MnO_2, as obtained with SRUP and FRUP.

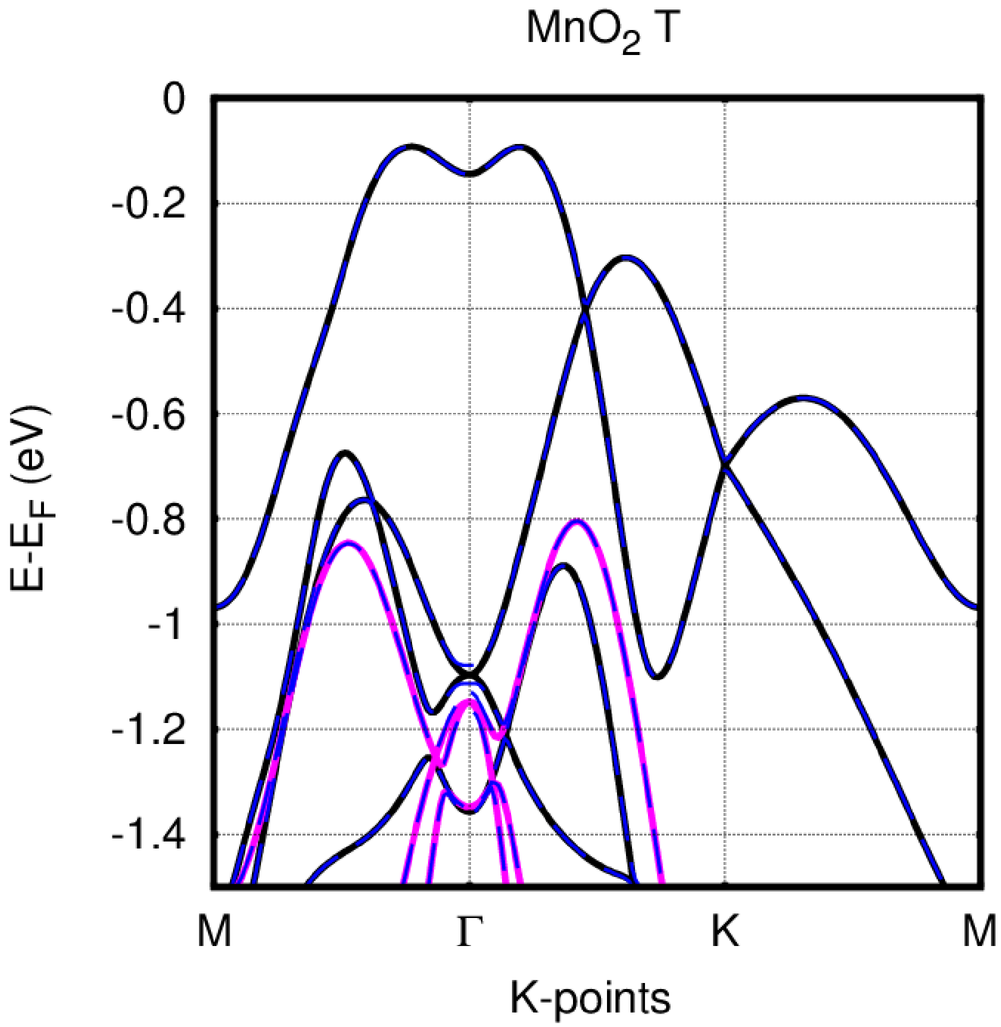

Supplementary 11. Zoom of electronic band structure for MnO_2, showing the splitting of the valance bands at the Γ point. Solid black, solid magenta and dashed blue lines represent spin up, spin down and non collinear bands, respectively. The inner bands, from -1.4 to -1 eV, present a bigger splitting in comparison with the maximum valence band.

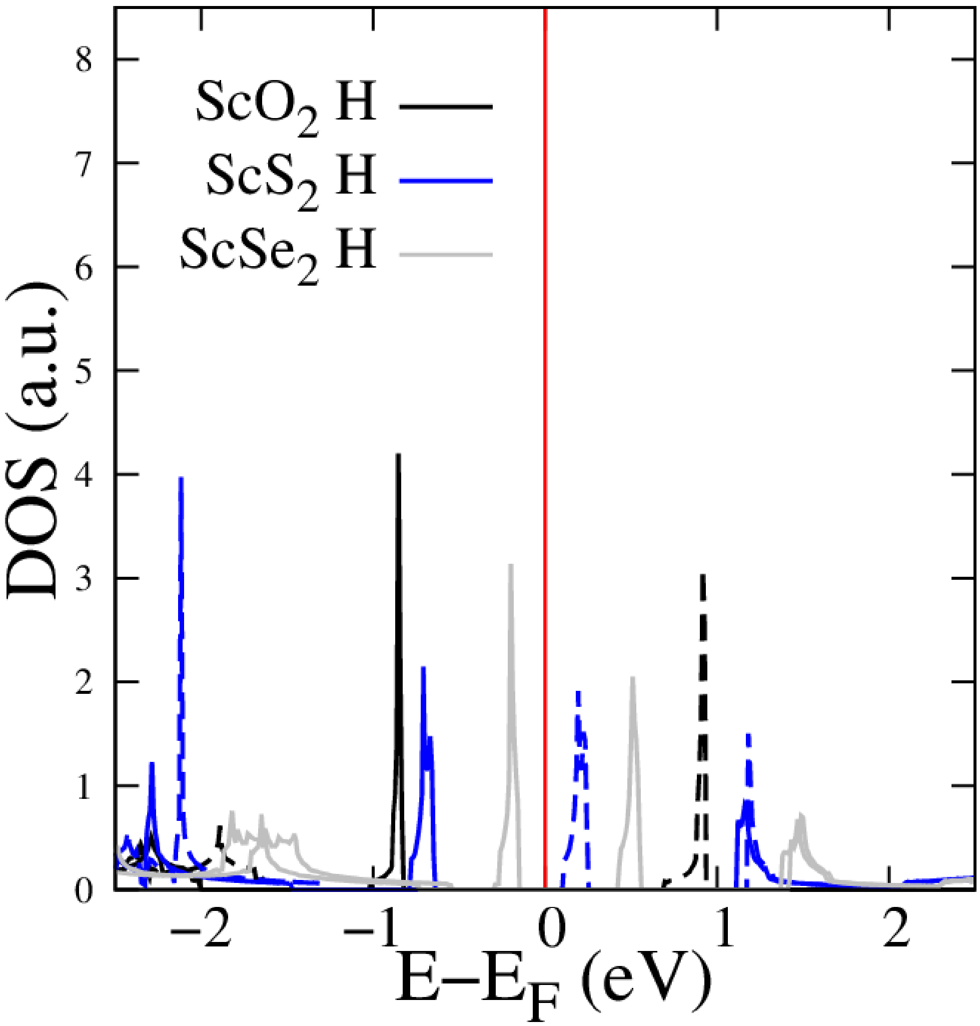

Supplementary 12. DOS of ScX_2, where X= O, S, Se and Te, as obtained with SRUP.

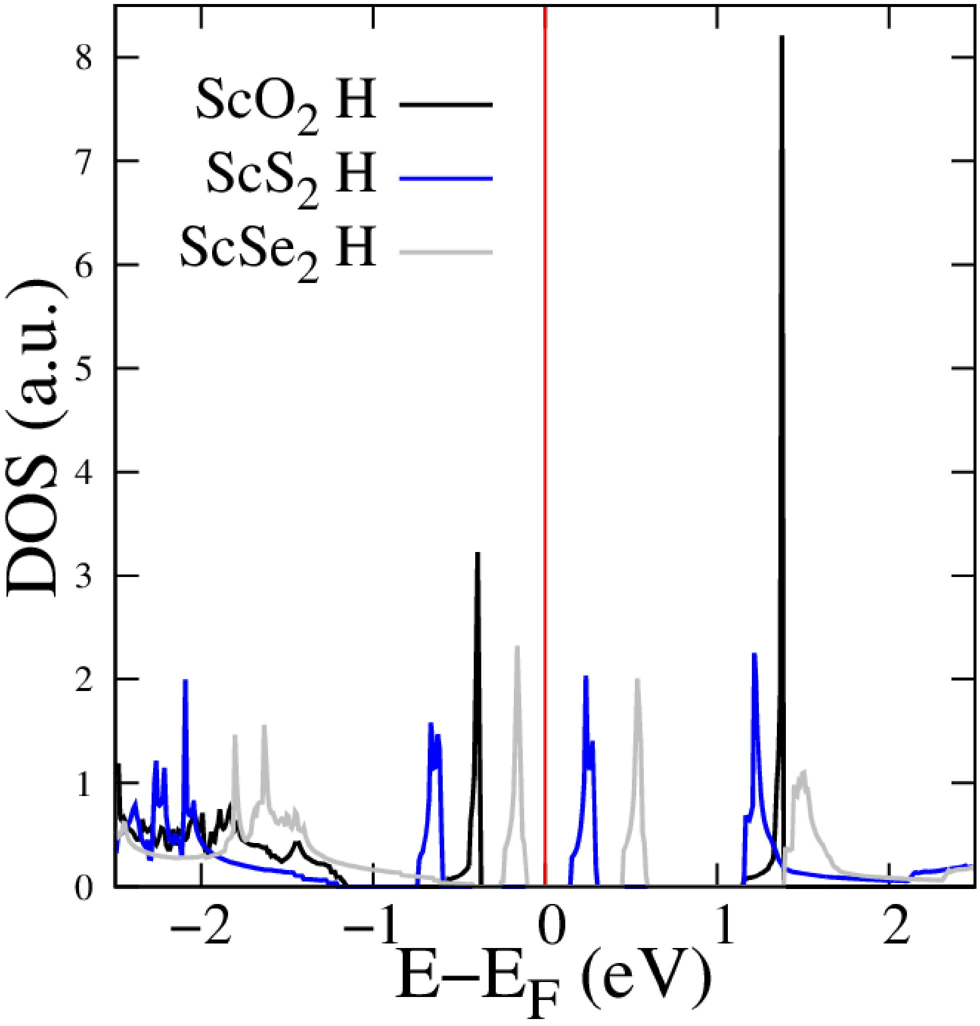

Supplementary 13. DOS of ScX_2, where X= O, S, Se and Te, as obtained with FRUP.

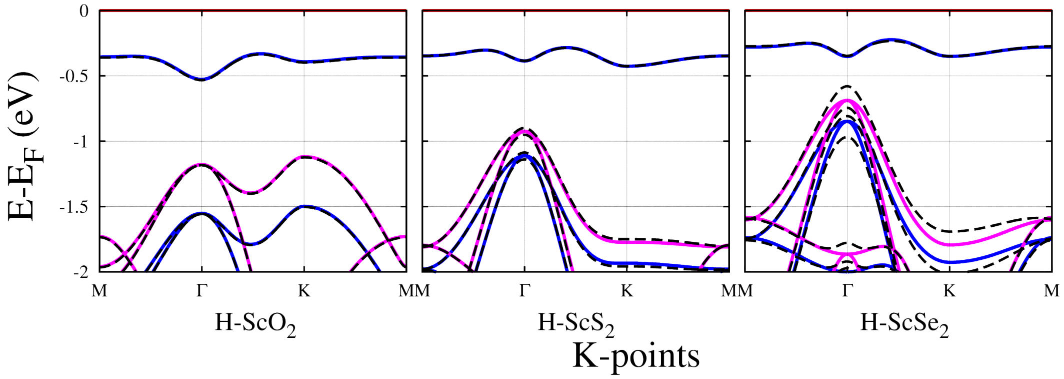

Supplementary 14. Electronic band structure of magnetic semiconductors 2D-MX_2. Solids (blue and magenta) and dash (black) lines represent the calculation without (spin up and spin down) and with spin orbit interaction.

WTe_2 H structure

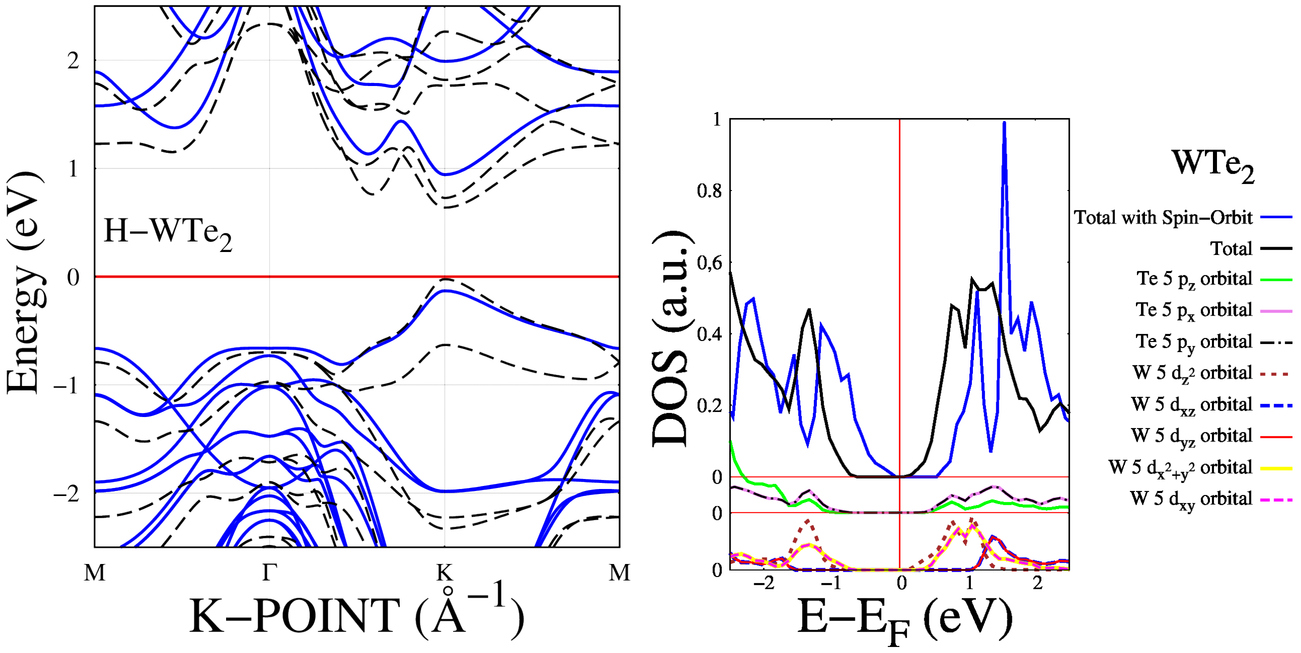

Supplementary 15. WTe_2 metastable semiconductor H structure. Left: Electronic band structure of nonmagnetic H-WTe_2 semiconductor calculated with spin orbit interaction (dash-black) and without it (solid-blue). Right: DOS and orbital-projected DOS for WTe_2. d_{z^2} (dash brown), $d_{x^2+y^2}$ (solid yellow) and d_{xy} (dash pink) are the main SRUP orbitals contributors from the VBM to the DOS (solid black). The major effect of the spin orbit reflects in the dispersion of these orbitals (solid blue). p and d orbitals are shifted for clarity.

Structures reported in the Inorganic Crystal Structure Data (ICSD)

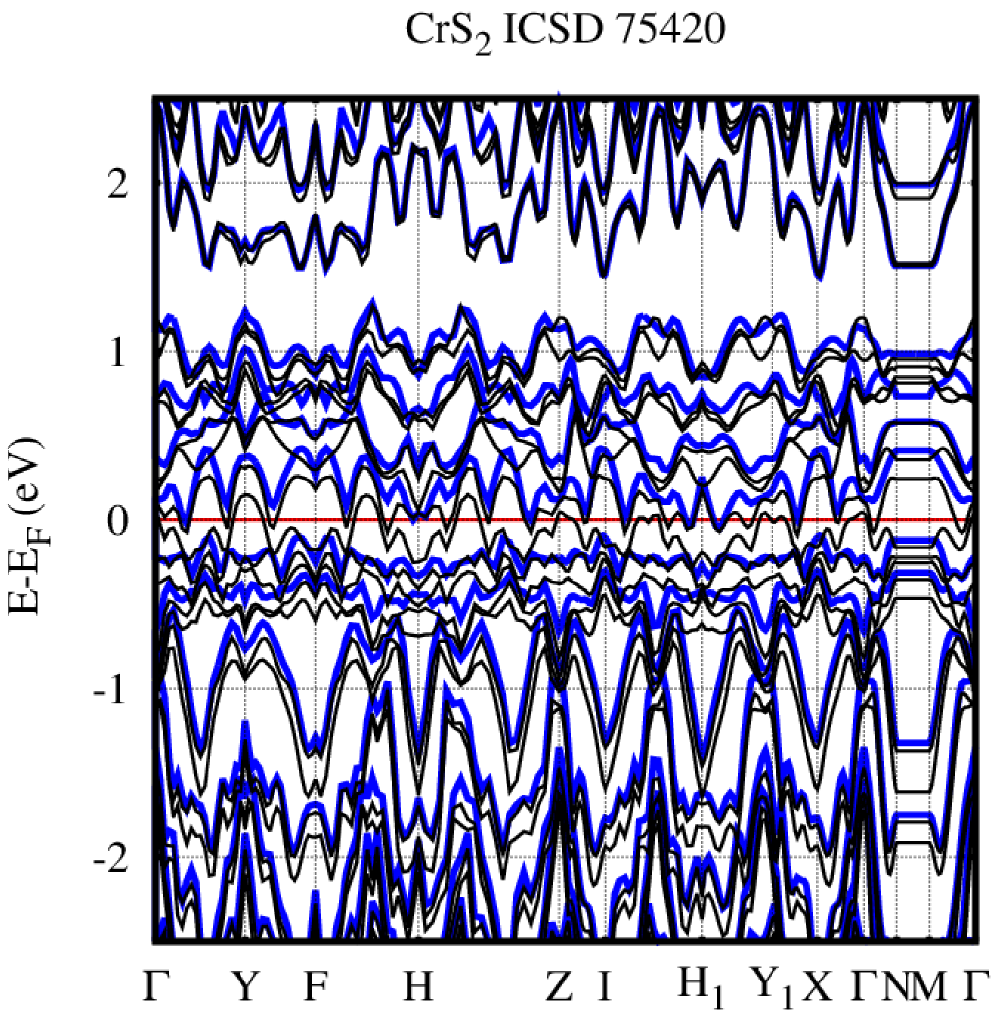

Supplementary 16. Band structure calculation of CrS_2. The initial structure of CrS_2 was taken from ICSD (75420) and then geometrical optimized. The wave vector path is taken from the suggestion of Ref.[1] The system is nonmagnetic and magnetic metallic (1.09 μ_B) when calculated with SRUP (blue) and FRUP (black) respectively. The highest occupied band has splitting with a value of 144 meV at Γ point.

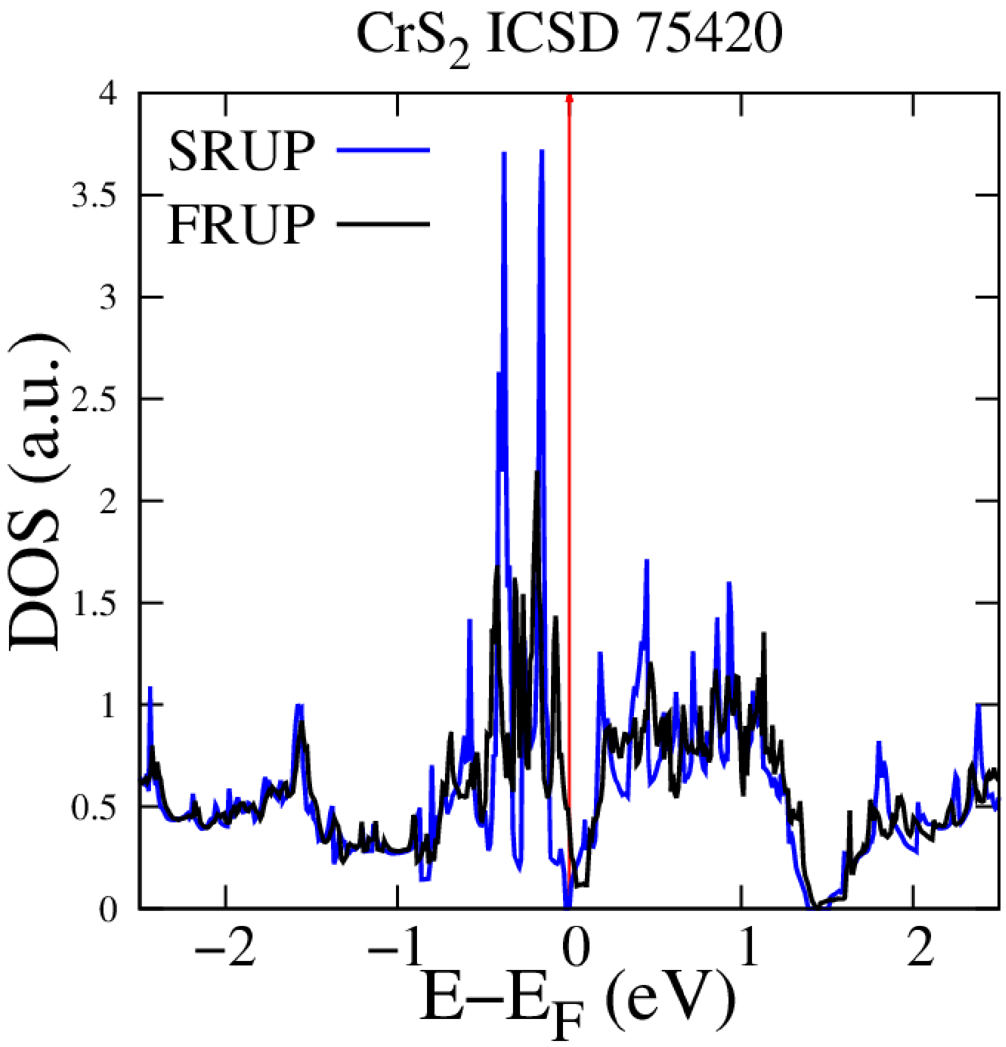

Supplementary 17. DOS of CrS_2, as obtained with SRUP (blue) and FRUP (black).

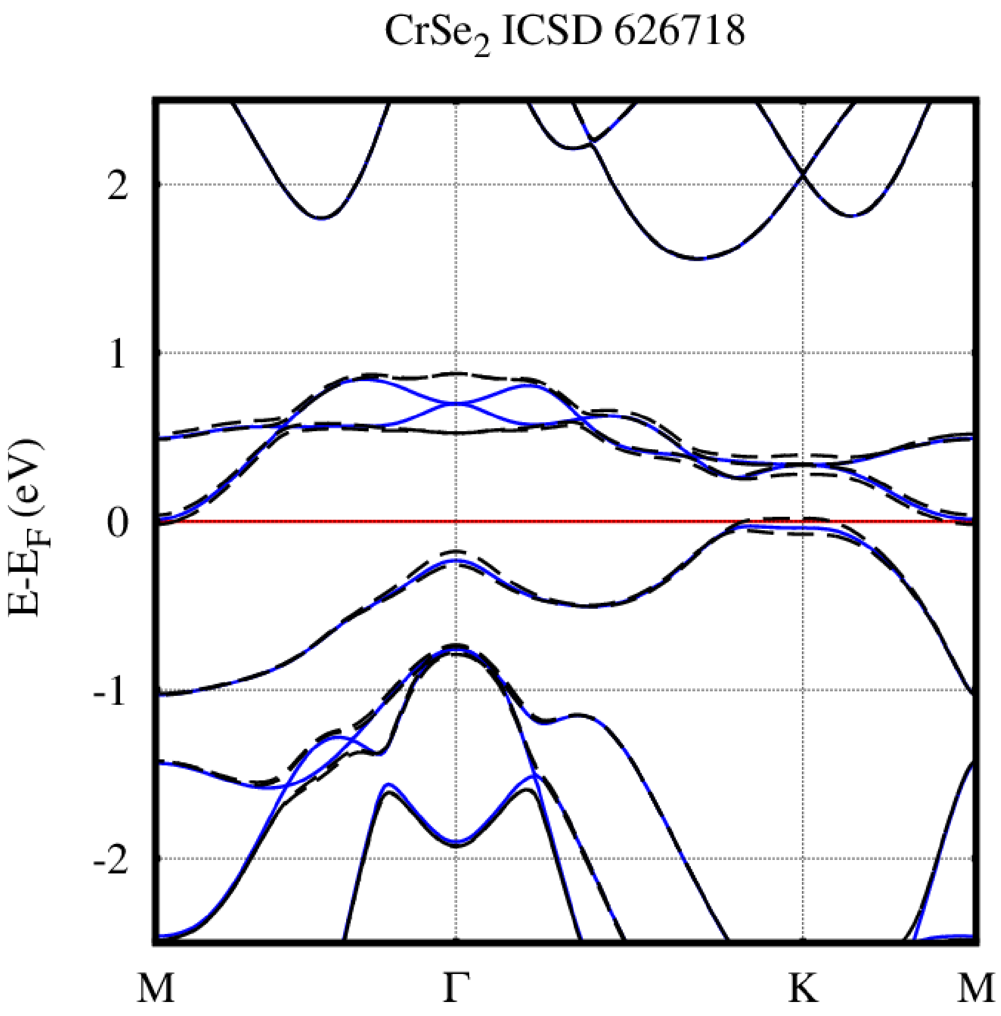

Supplementary 18. Band structure calculation of $CrSe_2$. The initial structure of CrS_2 was taken from ICSD (626718) and then geometrical optimized. The system is nonmagnetic and magnetic metallic (0.08 μ_B) when calculated with SRUP (blue) and FRUP (black) respectively. The highest occupied band has splittings with values of 80 and 91 meV at Γ and *K* point, respectively.

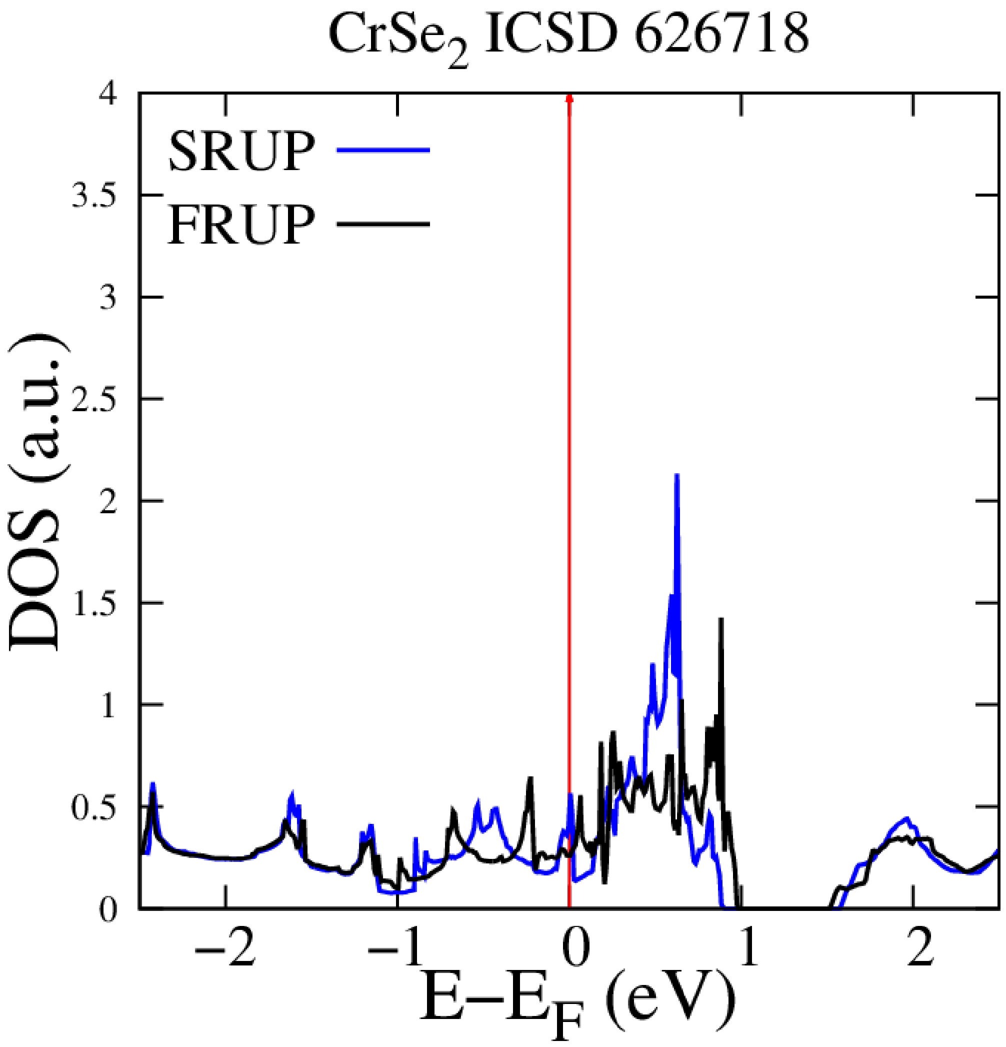

Supplementary 19. DOS of $CrSe_2$, as obtained with SRUP (blue) and FRUP (black).

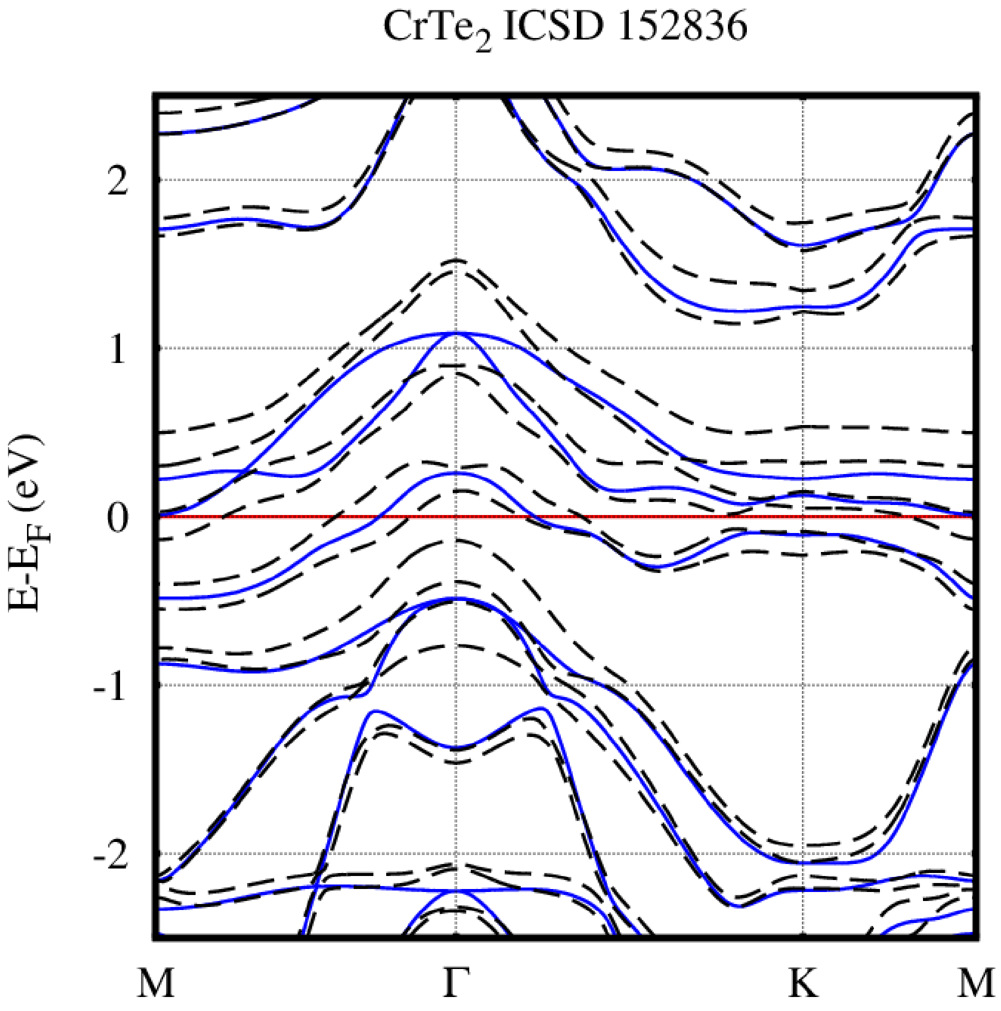

Supplementary 20. Band structure calculation of $CrTe_2$. The initial structure of $CrSe_2$ was taken from ICSD (152836) and then geometrical optimized. The system is nonmagnetic and magnetic metallic (0.33 μ_B) when calculated with SRUP (blue) and FRUP (black) respectively. The highest occupied band has splittings with values of 147 and 140 meV at Γ and K point, respectively.

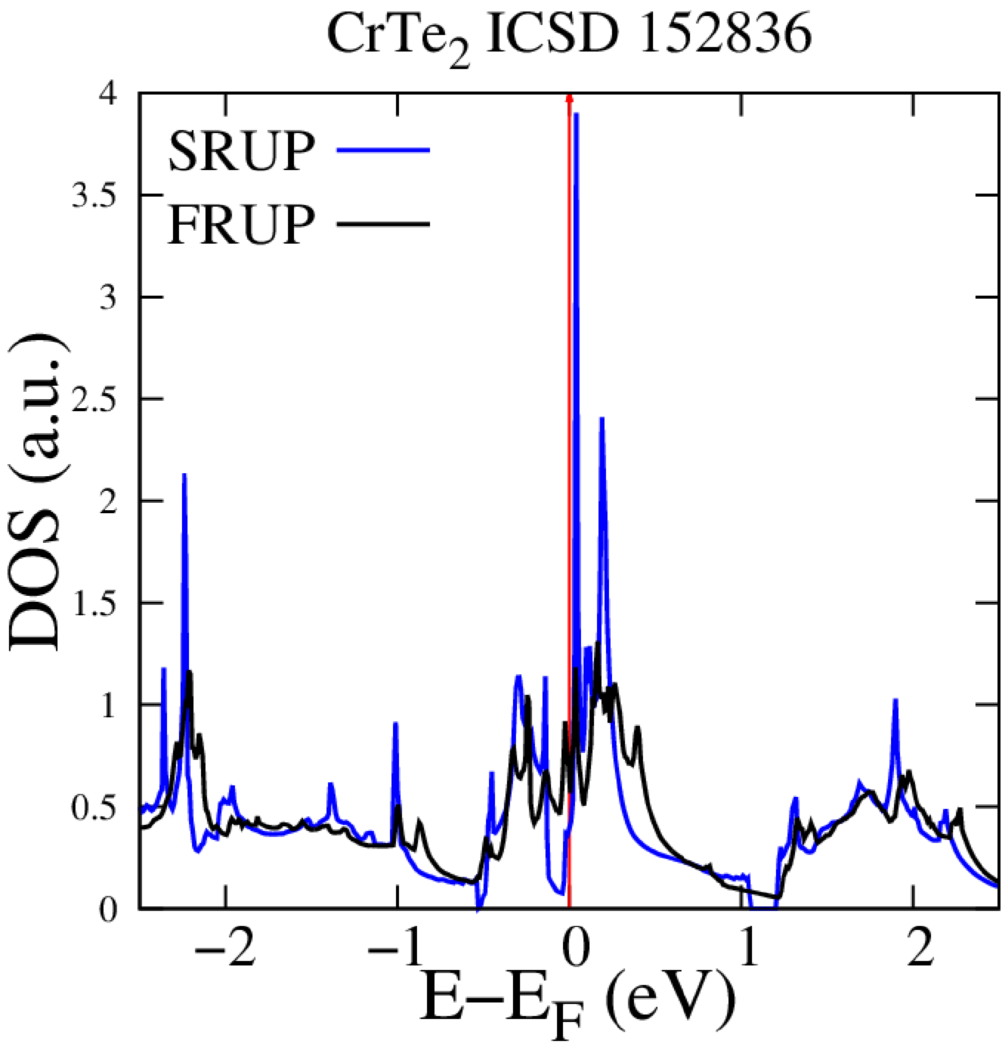

Supplementary 21. DOS of $CrTe_2$, as obtained with SRUP(blue) and FRUP (black)

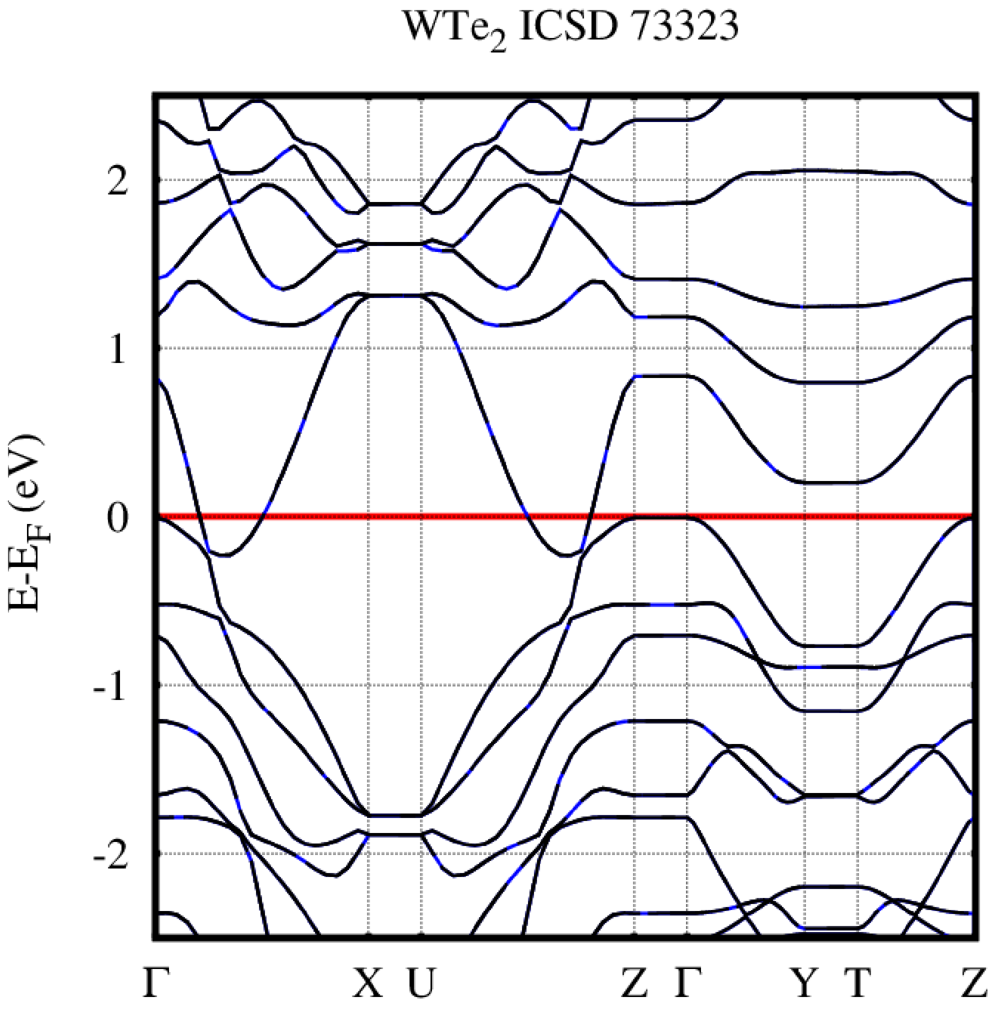

Supplementary 22. Band structure calculation of WTe_2. The initial structure of WTe_2 was taken from ICSD (73323) and then geometrical optimized. The system is metallic with both approximations: SRUP (blue) and FRUP (black).

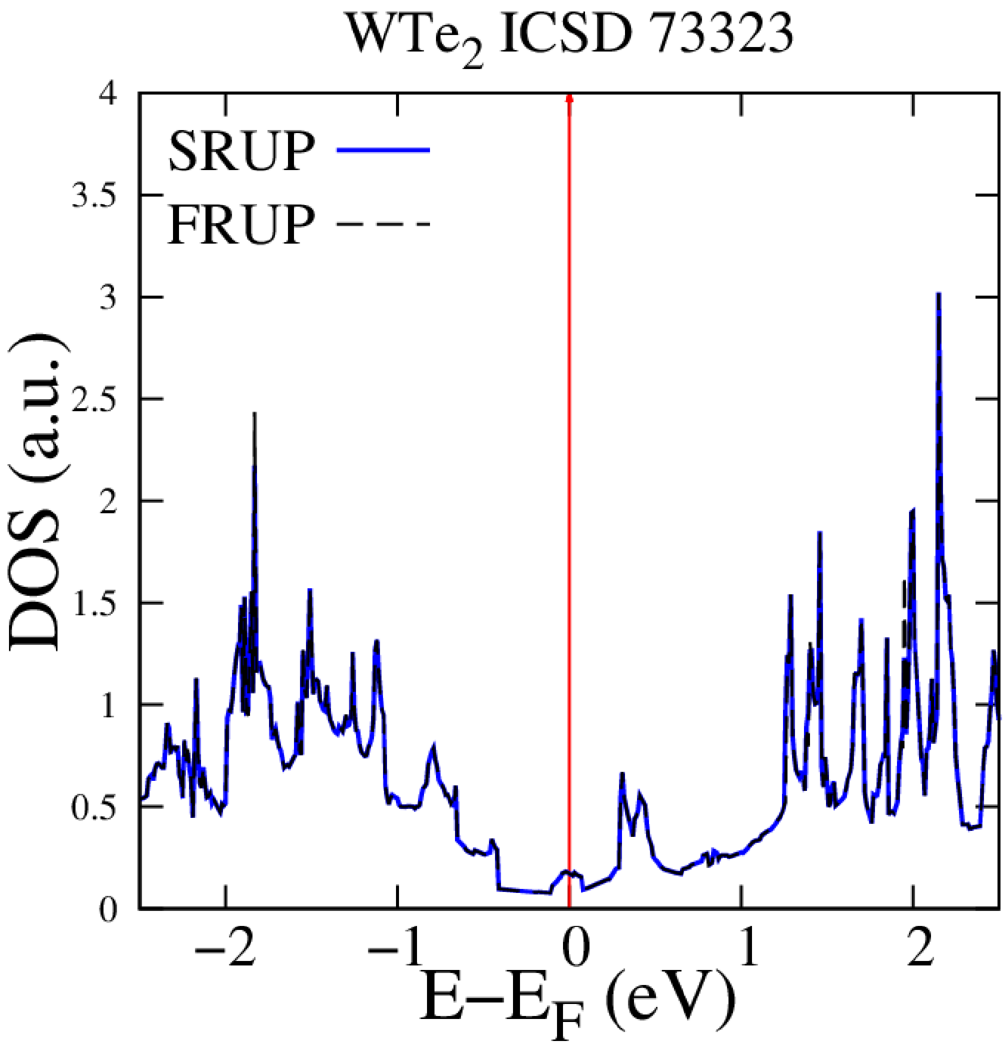

Supplementary 23. DOS of WTe_2, as obtained with SRUP (blue) and FRUP (black).

Atomic coordinates In this section, the relaxed atomic positions are presented. For those systems which 3D crystals exist, their ICSD number are included. Furthermore, the crystal coordinates are taken in order to build the monolayer. When the coordinates are indexed with **Relaxed**, the structure corresponds to the relaxed hexagonal structure with $a = b$ and a a distance of 10 Å between layers. Otherwise, for the ICSD data, the crystal structure is explicitly provided.

ScX_2

ScO_2

Relaxed

a=3.22 Å

Atomic coordinates (Å)

Sc 0.000 1.8610 0.000

O 1.612 0.9307 1.034

O 1.612 0.9307 -1.034

ScS_2

Relaxed a=3.79 Å

Atomic coordinates (Å)

Sc 0.000 2.188 0.000

S 1.895 1.094 1.356

S 1.895 1.094 -1.356

$ScSe_2$

Relaxed

a=3.95 Å

Atomic coordinates (Å)

Sc 0.000 2.281 0.000

Se 1.975 1.140 1.465

Se 1.975 1.140 -1.465

CrX_2

CrO_2

Relaxed

a=2.63 Å

Atomic coordinates (Å)

Cr 0.000 1.5160 0.000

O 1.313 0.7579 1.156

O 1.313 0.7579 -1.156

CrS_2

ICSD 75420

a=3.46 Å

b=5.79Å

The distance between layers is 10 Å

α= 95.54

Atomic coordinates (Å)

S -0.17587 1.81319 -1.36305

S 1.27480 4.69310 -1.36305

S -0.38280 3.94663 1.36305

S 1.62653 1.06672 1.36305

Cr 0.00000 0.00000 0.00000

Cr 1.45067 2.87991 0.00000

ICSD 75420 after geometrical relaxation

a=3.18 Å

b=6.10 Å

The distance between layers is 10 Å

α= 95.54

Atomic coordinates (Å)

S -0.16151 1.66520 -1.25180

S 1.17075 4.31006 -1.25180

S -0.35156 3.62451 1.25180

S 1.49378 0.97965 1.25180

Cr 0.00000 0.00000 0.00000

Cr 1.33226 2.64486 0.00000

Relaxed

a=3.05 Å

Atomic coordinates (Å)

Cr 0.000 1.7590 0.000

S 1.524 0.8796 1.464

S 1.524 0.8796 -1.464

$CrSe_2$

ICSD 626718

a=3.40 Å

The distance between layers is 10 Å

Atomic coordinates (Å)

Cr 0.00000 0.00000 0.00000

Se 0.00000 1.96240 1.47872

Se 1.69949 0.98120 -1.47872

ICSD 626718 after geometrical relaxation

a=3.31 Å

The distance between layers is 10 Å

Atomic coordinates (Å)

Cr 0.000 0.000 0.000

Se 0.000 1.911 1.440

Se 1.655 0.955 -1.440

Relaxed

a=3.22 Å

Atomic coordinates (Å)

Cr 0.000 1.8570 0.000

Se 1.608 0.9285 1.571

Se 1.608 0.9285 -1.571

$CrTe_2$

ICSD 152836

This ICSD corresponds to $TlCrTe_2$. The thallium atoms have been intentionally removed in order to build the T CrTe2. a=4.02 Å

The distance between layers is 10 Å
Cr 0.000 0.000 3.968
Te 0.000 2.318 2.507
Te 2.008 1.159 -2.507

ICSD 152836 after geometrical relaxation
This ICSD corresponds to $TlCrTe_2$. The thallium atoms have been intentionally removed in order to build the *T* CrTe2. a=3.73 Å
The distance between layers is 10 Å
Cr 0.000 0.000 3.686
Te 0.000 2.154 2.329
Te 1.865 1.077 -2.329

Relaxed a=3.48 Å
Atomic coordinates (Å)
Cr 0.000 2.007 0.000
Te 1.738 1.003 1.706
Te 1.738 1.003 -1.706

MnO_2
Relaxed
a=2.96 Å
Atomic coordinates (Å)
Mn 1.482 0.8558 -0.9514
O 0.000 1.7120 -1.9030
O 0.000 0.0000 0.0000

NiX_2
NiO_2
Relaxed
a=2.86 Å
Atomic coordinates (Å)
Ni 1.43273 0.82719 -0.95864
O 0.00000 1.65437 -1.91729
O 0.00000 0.00000 0.00000

NiS_2
Relaxed
a=3.33 Å
Atomic coordinates (Å)
Ni 1.66387 0.96064 -1.18092
S 0.00000 1.92128 -2.36189
S 0.00000 0.00000 0.00000

$NiSe_2$
Relaxed
a=3.51 Å
Atomic coordinates (Å)
Ni 1.75280 1.01198 -1.27299
Se 0.00000 2.02396 -2.54599
Se 0.00000 0.00000 0.00000

MoX_2
MoO_2
Relaxed
a=2.84 Å
Atomic coordinates (Å)
Mo 0.00000 1.64017 0.00000
O 1.42042 0.82008 1.23428
O 1.42042 0.82008 -1.23428

MoS_2
ICSD 644254
a=3.16 Å
The distance between layers is 10 Å
Atomic coordinates (Å)
Mo 0.91293 1.58125 3.07555
S 1.82587 0.00000 4.65638
S 1.82587 0.00000 1.49472

Relaxed
a=3.20 Å
Atomic coordinates (Å)
Mo 0.00000 1.84679 0.00000
S 1.59937 0.92339 1.55911
S 1.59937 0.92339 -1.55911

$MoSe_2$
ICSD 644334
a=3.29 Å
The distance between layers is 10 Å
Atomic coordinates (Å)
Mo 0.94916 1.64400 3.22500
Se 1.89833 0.00000 4.83750
Se 1.89833 0.00000 4.83750

Relaxed
a=3.33 Å
Atomic coordinates (Å)
Mo 0.00000 1.92299 0.00000
Se 1.66536 0.96149 1.67279
Se 1.66536 0.96149 -1.67279

$MoTe_2$
ICSD 15431
a=3.52 Å
The distance between layers is 10 Å
Atomic coordinates (Å)
Mo 1.01585 1.75950 3.49100
Te 2.03170 0.00000 5.23650
Te 2.03170 0.00000 1.74550

Relaxed
a=3.55 Å
Atomic coordinates (Å)
Mo 0.00000 2.04853 0.00000

Te 1.77408 1.02426 1.81336
Te 1.77408 1.02426 -1.81336

WX_2

WO_2

Relaxed

a=2.83 Å
Atomic coordinates (Å)
W 0.00000 1.63720 0.00000
O 1.41786 0.81860 1.23579
O 1.41786 0.81860 -1.23579

WS_2

ICSD 202366

a=3.15 Å
The distance between layers is 10 Å
Atomic coordinates (Å)
W 0.91025 1.57660 3.08075
S 1.82050 0.00000 4.65193
S 1.82050 0.00000 1.50957

Relaxed

a=3.19 Å
Atomic coordinates (Å)
W 0.00000 1.84312 0.00000
S 1.59619 0.92156 1.56567
S 1.59619 0.92156 -1.56567

WSe_2

ICSD 40752

a=3.28 Å
The distance between layers is 10 Å
Atomic coordinates (Å)
W 0.94743 1.64100 3.24000
Se 1.89486 0.00000 4.91054
Se 1.89486 0.00000 1.56946

Relaxed

a=3.33 Å
Atomic coordinates (Å)
W 0.00000 1.92042 0.00000
Se 1.66313 0.96021 1.67874
Se 1.66313 0.96021 -1.67874

WTe_2

ICSD 73323

a=3.47 Å
b=6.25 Å
The distance between layers is 10 Å
Atomic coordinates (Å)
Te 0.000000000 5.359139218 9.185182184
Te 0.000000000 1.294902064 5.661380005
Te 1.738478739 4.383932268 5.044161988
Te 1.738478739 2.210181914 8.566575122
W 1.738478739 6.000216466 7.222265492
W 0.000000000 3.753228523 7.008914166

ICSD 73323 after geometrical relaxation

a=3.47 Å
b=6.32 Å
The distance between layers is 10 Å
Atomic coordinates (Å)
Te 0.000000000 5.427947354 9.198842837
Te 0.000000000 1.320063145 5.597634523
Te 1.729329861 2.210494267 8.594200608
Te 1.729329861 4.405277269 8.974162489
W 0.000000000 3.794891687 6.997644483
W 1.729329861 6.037553554 7.194448933

Relaxed

a=3.56 Å
Atomic coordinates (Å)
W 0.00000 2.05601 0.00000
Te 1.78056 1.02801 1.81232
Te 1.78056 1.02801 -1.81232

References

1. Setyawan, W. & Curtarolo, S. High-throughput electronic band structure calculations: Challenges and tools. *Computational Materials Science* **49**, 299–312 (2010).
